# Therapy as a State-Generator: Dynamic Phenotypic Landscapes and Adaptive Stress Circuits in Chemotherapy Resistance of Breast Cancer

**DOI:** 10.3390/antiox15040459

**Published:** 2026-04-08

**Authors:** Moon Nyeo Park

**Affiliations:** College of Korean Medicine, Kyung Hee University, 1-5 Hoegidong, Dongdaemun-gu, Seoul 02447, Republic of Korea; mnpark@khu.ac.kr

**Keywords:** breast cancer, chemotherapy resistance, redox signaling, integrated stress response, metabolic reprogramming, phenotypic plasticity

## Abstract

Chemotherapy resistance remains a major obstacle to durable cancer control, yet its underlying mechanisms cannot be fully explained by genetic mutations alone. Increasing evidence suggests that therapeutic stress induces dynamic adaptive programs that reshape tumor phenotypic landscapes. Here, we propose a systems-level framework in which chemotherapy resistance emerges from the stabilization of interconnected stress-response circuits integrating redox signaling, metabolic reprogramming, and transcriptional plasticity. In this model, cytotoxic therapies function as state-generating perturbations that elevate oxidative stress and activate adaptive buffering systems, including NADPH-dependent redox homeostasis, replication stress tolerance, and integrated stress response (ISR)-mediated translational reprogramming. These adaptive modules collectively expand the accessibility of therapy-tolerant phenotypic states within tumor cell populations. Importantly, these circuits coordinate mitochondrial redox homeostasis, metabolic NADPH regeneration, and epigenetic–transcriptional plasticity to sustain cellular survival under persistent oxidative pressure. Such adaptive redox networks not only stabilize stress-tolerant phenotypes but also create vulnerabilities that can be therapeutically exploited. From a translational perspective, this framework suggests that effective strategies to overcome chemotherapy resistance should move beyond single-target inhibition and instead focus on circuit-guided therapeutic interventions that simultaneously destabilize redox buffering systems, constrain phenotypic plasticity, and disrupt metabolic stress adaptation. By conceptualizing therapy resistance as a dynamic redox-regulated state-space phenomenon, this model provides a mechanistic foundation for the development of evolution-aware and plasticity-constraining therapeutic strategies. Targeting the coordinated redox–metabolic–translational circuits that maintain tumor adaptability may therefore represent a promising direction for next-generation redox therapeutics in cancer.

## 1. Introduction

Breast cancer remains a biologically heterogeneous disease characterized by distinct molecular subtypes, including estrogen receptor-positive (ER^+^), Human Epidermal growth factor Receptor 2-positive (HER2^+^), and triple-negative breast cancer (TNBC). Despite advances in subtype-specific therapies, therapeutic resistance—both endocrine and chemotherapy resistance—continues to represent a major clinical challenge. Increasing evidence suggests that resistance is not solely driven by isolated genetic alterations, but rather emerges from dynamic adaptive survival programs orchestrated by steroid receptor signaling networks and downstream stress-response circuitry [1,2,3]. Intratumoral heterogeneity plays a foundational role in therapeutic failure. In HER2^+^ disease, variability in receptor expression, intracellular trafficking, immune microenvironment composition, and signaling plasticity contributes to incomplete responses to targeted agents and antibody–drug conjugates [1]. Similarly, ER-low tumors introduce diagnostic and biological ambiguity, with repeated molecular reassessment frequently reclassifying cases as ER-null, underscoring the instability of receptor-defined categories and their therapeutic implications [4]. These observations collectively emphasize that receptor status alone is insufficient to predict long-term therapeutic vulnerability. In ER-positive disease, endocrine resistance frequently arises through Estrogen Receptor 1 (ESR1) ligand-binding domain mutations, alternative pathway activation, and PI3K/AKT signaling engagement [2]. These molecular adaptations allow tumor cells to sustain proliferative and survival signaling independent of estrogen availability. Importantly, many of these adaptive pathways overlap with those implicated in chemotherapy resistance, suggesting convergence upon conserved stress-survival modules. Clinically, once endocrine resistance develops, therapeutic options narrow substantially, often defaulting to sequential chemotherapy with modest response rates and cumulative toxicity [5]. This therapeutic bottleneck reflects a critical unmet need. Beyond canonical genomic signaling, steroid receptors—including ER, androgen receptor (AR), and glucocorticoid receptor (GR)—also engage extranuclear and rapid signaling mechanisms that activate Src, PI3K, ERK, and Akt pathways [3]. These non-genomic circuits potentiate cellular survival under cytotoxic stress and may facilitate adaptive tolerance to chemotherapy. Thus, steroid receptor signaling functions not only as a lineage-defining driver but also as a stress-adaptive regulatory platform. Recent clinical advances, such as AKT inhibition in endocrine-resistant settings [6] and antibody–drug conjugates in heavily pretreated HR^+^/HER2^−^ disease [5], demonstrate that targeting downstream adaptive nodes can improve outcomes. However, resistance inevitably re-emerges, highlighting the limitations of single-axis targeting strategies and the need for circuit-based therapeutic design. Taken together, breast cancer resistance should be conceptualized not merely as receptor escape or clonal mutation, but as the activation of integrated adaptive survival programs. This review proposes a circuit-based framework linking steroid receptor signaling to conserved adaptive modules that drive chemotherapy resistance and outlines biomarker-guided combination strategies aimed at overcoming these mechanisms. To clearly distinguish adaptive regulatory circuits from linear signaling pathways, we define a circuit-level regulatory module as a feedback-governed network architecture that incorporates self-reinforcing or reciprocal feedback interactions, maintains phenotypic persistence or memory following transient perturbations, and generates measurable functional outputs such as stable cell-state transitions or resistance phenotypes [7,8]. Such circuits are fundamentally distinct from linear pathways in that their behavior emerges from non-linear network motifs—including feedback loops and feedforward structures—that enable state stabilization, hysteresis, and dynamic adaptability under therapeutics stress [9,10].

### 1.1. Steroid Receptor-Driven Adaptive Transcription as the Upstream Hub

Steroid receptors function not only as lineage-defining transcription factors but also as dynamic integrators of stress-adaptive signaling. In breast cancer, estrogen receptor (ER), androgen receptor (AR), and glucocorticoid receptor (GR) operate within a highly plastic transcriptional network that undergoes context-dependent rewiring under therapeutic pressure [11]. Rather than acting as static oncogenic drivers, these receptors coordinate adaptive transcriptional programs that enable phenotypic redistribution within a constrained state-space. A central mechanism underlying this plasticity is ER–cofactor rewiring. Steroid receptor activity is critically dependent on coregulator complexes, particularly the Steroid Receptor Coactivator (SRC) family and associated chromatin-modifying enzymes. Altered recruitment of coactivators and corepressors reshapes enhancer landscapes, modifies chromatin accessibility, and reprograms downstream transcriptional outputs independent of changes in receptor abundance [12]. Ligand-independent receptor activation, frequently observed in endocrine-resistant tumors, further amplifies this adaptive flexibility by sustaining transcriptional signaling despite therapeutic blockade [13]. Steroid receptor rewiring also biases DNA repair and replication programs. ER and GR signaling influence transcription of genes involved in stress adaptation and genomic regulation, including context-dependent assembly of transcriptional regulatory factors and chromatin remodeling complexes [14]. Such transcriptional shifts enhance tolerance to replication stress and genotoxic injury, thereby facilitating survival following chemotherapy-induced DNA damage. Concurrently, steroid receptor signaling induces metabolic reprogramming. Through direct transcriptional regulation of mitochondrial respiratory genes and oxidative phosphorylation components, nuclear steroid receptors coordinate mitochondrial function and bioenergetic output [15]. GR-mediated metabolic control further integrates glucose, amino acid, and lipid metabolism with transcriptional programs in peripheral tissues [16]. In endocrine-resistant models, combined ERα and XPO1 targeting demonstrates that receptor rewiring is tightly coupled to metabolic pathway remodeling, including Akt signaling and mitochondrial dependency [13]. Steroid receptor signaling also intersects with xenobiotic metabolism and steroid-processing enzymes, influencing cytochrome P450-mediated steroid turnover and transcriptional adaptation [17]. Alterations in glucocorticoid receptor expression and corticosteroid metabolism have been directly associated with differential therapeutic responsiveness, underscoring the clinical relevance of receptor-driven transcriptional remodeling [18]. Collectively, steroid receptor-driven adaptive transcription operates as an upstream regulatory hub that functionally couples to downstream modules, including redox–ferroptosis threshold regulation, DDR–replication stress tolerance, integrated stress response activation, and microenvironmental stabilization. This hub-centric framework reframes endocrine and chemotherapy resistance as coordinated transcriptional state transitions rather than isolated pathway escape events, providing a mechanistically grounded and clinically actionable foundation for coupling-target therapeutic strategies.

### 1.2. DDR–Replication Stress Tolerance

DNA replication stress (RS) represents a central adaptive bottleneck in therapy-exposed breast cancer cells. Cytotoxic chemotherapy, endocrine therapy, and oncogene-driven hyperproliferation converge on replication fork instability, generating stalled or collapsed forks that threaten genomic integrity [19]. In this context, activation of the ATR–CHK1 signaling axis functions as a core survival buffer, stabilizing replication forks, limiting excessive origin firing, and coordinating S-phase checkpoint responses [20]. Oncogene activation—including MYC, cyclin E, and RAS—induces intrinsic replication stress by accelerating origin firing and replication fork progression, thereby increasing the burden of stalled forks and single-stranded DNA accumulation [21]. Rather than collapsing under this stress, cancer cells develop replication stress tolerance (RST), in which ATR-mediated signaling suppresses lethal double-strand break formation while preserving fork stability [22]. This tolerance permits continued proliferation despite chronic genomic instability, thereby fueling tumor evolution. Mechanistically, ATR–CHK1 signaling regulates fork speed, origin firing, and homologous recombination-mediated repair pathways [23]. CHK1 suppresses excessive replication origin activation and promotes fork stabilization by coordinating CtIP-, BRCA1-, and HR-dependent repair processes [24]. In radioresistant or chemotherapy-exposed breast cancer cells, elevated ATR/CHK1 activity reflects dependency on replication stress buffering for survival [25]. Importantly, replication fork remodeling has emerged as a critical determinant of therapy escape. Under stress, forks undergo controlled reversal and protection mediated by RAD51 and associated factors. Loss of fork protection leads to nascent strand degradation and chemosensitivity, whereas restoration of fork stability contributes to acquired resistance. Thus, therapy resistance in DDR-deficient tumors may arise not solely from homologous recombination restoration but from replication fork stabilization independent of canonical HR repair [26,27]. Single-cell analyses further demonstrate that replication stress can induce ATR–CHK1-dependent asymmetric chromatid segregation, promoting survival of stress-adapted daughter cells while eliminating heavily damaged populations [28]. This dynamic segregation behavior illustrates that DDR buffering is not merely repair-centric but contributes to state redistribution within tumor populations. Collectively, DDR–replication stress tolerance constitutes a dynamic buffering module within the SR^3^ network. Rather than functioning solely as a repair pathway, ATR–CHK1 signaling operates as a stress-threshold regulator that permits continued proliferation under genotoxic and oncogenic stress. Therapeutic strategies targeting this module must therefore account for the adaptive reprogramming capacity of replication fork remodeling and checkpoint signaling rather than viewing DDR inhibition as a simple synthetic lethal intervention.

### 1.3. ISR-Translational Plasticity

The integrated stress response (ISR) functions as a translational rheostat that enables breast cancer cells to dynamically recalibrate proteostasis under therapeutic and metabolic stress. Central to this mechanism is phosphorylation of eukaryotic translation initiation factor 2 alpha (eIF2α), which suppresses global cap-dependent translation while selectively enhancing translation of stress-adaptive transcripts such as activating transcription factor 4 (ATF4) [29]. Phosphorylation of eIF2α inhibits its guanine nucleotide exchange factor eIF2B, reducing formation of the eIF2–GTP–Met-tRNA ternary complex and thereby attenuating bulk protein synthesis [30]. Paradoxically, this translational repression permits preferential translation of mRNAs containing inhibitory upstream open reading frames (uORFs), including ATF4, thereby reprogramming the proteome toward stress adaptation [31]. ATF4 subsequently orchestrates transcriptional programs governing amino acid metabolism, redox balance, autophagy, and apoptotic signaling [29]. Recent work demonstrates that ISR signaling exhibits substantial plasticity rather than functioning as a linear binary switch. A non-canonical ISR branch termed the “split ISR” (s-ISR) can be activated by attenuation of eIF2B activity independent of eIF2α phosphorylation, relying instead on eIF4E-dependent translational control of ATF4 and metabolic rewiring pathways [32]. This plastic architecture suggests that ISR outputs vary according to stress intensity, duration, and upstream signaling context. ISR plasticity is further governed by regulatory phosphatase circuits. PP2A-mediated modulation of ISR signaling has been shown to determine whether stress responses remain reversible and adaptive or transition into irreversible cytotoxic programs [33]. In this model, normal cells exhibit intrinsic ISR reversibility, enabling recovery from chronic stress, whereas malignant cells display maladaptive ISR persistence, rendering them vulnerable to sustained stress amplification [34]. Beyond translational attenuation, ISR intersects with mitochondrial and proteotoxic stress pathways. Heme-regulated inhibitor (HRI) activation links cytosolic proteotoxicity and mitochondrial dysfunction to ISR induction, coordinating mitonuclear communication and proteostasis remodeling [35]. This connection reinforces the integration of ISR within the broader redox–metabolic stress axis described in Module 2. Importantly, translational reprogramming under ISR extends beyond ATF4 induction. Stress-dependent alterations in initiation complex assembly, scanning dynamics, and RNA-binding protein interactions collectively reshape translation efficiency across subsets of transcripts [36]. Such selective translation plasticity permits tumors to maintain synthesis of survival-promoting proteins despite global translational repression. Within the SR^3^ network framework, ISR–translation plasticity functions as a buffering module that converts redox imbalance, replication stress, and metabolic perturbation into adaptive proteomic states. When transient and reversible, ISR supports tumor survival under endocrine therapy, chemotherapy, and metabolic stress. When sustained beyond adaptive capacity, however, ISR signaling promotes apoptotic execution programs via ATF4–CHOP-dependent pathways [29]. Thus, ISR should not be conceptualized as merely a stress-response pathway, but rather as a state-transition regulator that governs the boundary between cytostatic adaptation and irreversible cell fate commitment. Targeting ISR plasticity may therefore offer a strategy to collapse stress-tolerant tumor states rather than solely suppressing upstream oncogenic drivers.

## 2. Clinical Landscape: Endocrine Resistance and Transition to Chemotherapy

### 2.1. ER-Positive Breast Cancer

Endocrine therapy remains the backbone of treatment for ER-positive breast cancer. However, resistance frequently develops through ESR1 hotspot mutations (e.g., Y537, D538), ligand-independent receptor activation, and compensatory pathway engagement [37]. PI3K pathway activation, observed in approximately 40% of ER-positive tumors, further contributes to endocrine escape and disease progression [2]. As endocrine resistance evolves, tumors increasingly rely on stress-response and survival pathways that overlap mechanistically with chemotherapy resistance. Clinically, patients progressing on endocrine therapy—often combined with CDK4/6 inhibitors—are commonly transitioned to cytotoxic chemotherapy [38]. Yet response rates in this setting are modest, and cumulative toxicity limits sustained disease control [5]. This transition underscores a fundamental therapeutic gap: endocrine resistance often precedes, and mechanistically primes, chemotherapy resistance.

### 2.2. HER2-Positive Disease

Although HER2-targeted therapies have dramatically improved outcomes, resistance arises through receptor heterogeneity, altered internalization dynamics, lysosomal processing variability, and compensatory signaling activation [1]. Antibody–drug conjugates partially address this challenge; however, resistance mechanisms—including efflux transporter upregulation and pathway reactivation—remain significant barriers [39]. These findings reinforce the notion that adaptive signaling circuitry, rather than isolated target loss, drives therapeutic escape.

### 2.3. Triple-Negative Breast Cancer

TNBC is intrinsically reliant on chemotherapy. Yet neoadjuvant resistance and residual disease are common, with steroid receptor components such as GR and AR implicated in survival signaling under cytotoxic stress [3]. The absence of a dominant targetable receptor in TNBC highlights the importance of understanding shared adaptive modules that sustain survival during chemotherapy exposure.

#### 2.3.1. Clinical Context: Where Chemotherapy Resistance Emerges in Breast Cancer

Breast cancer chemotherapy resistance does not arise as an isolated pharmacologic failure but rather emerges from subtype-specific adaptive trajectories shaped by receptor signaling, microenvironmental remodeling, clonal evolution, and metabolic plasticity. (Across ER^+^, HER2^+^, and TNBC subtypes, resistance to cytotoxic therapy frequently represents the culmination of earlier adaptive escape programs that progressively rewire survival circuitry under therapeutic pressure.) Understanding these evolutionary pathways is essential for contextualizing the redox–ISR–plasticity modules discussed in subsequent sections.

#### 2.3.2. ER^+^ Breast Cancer: From Endocrine Escape to Chemotherapy Resistance

Estrogen receptor-positive (ER^+^) breast cancer accounts for approximately 70–80% of newly diagnosed cases and is characterized by initial responsiveness to endocrine therapy yet a persistent long-term risk of recurrence, extending up to two decades after primary treatment [40]. Unlike triple-negative breast cancer (TNBC), which typically recurs within the first 3–5 years, ER^+^ tumors frequently exhibit late relapse, reflecting a biology tightly linked to dormancy, adaptive signaling rewiring, and progressive therapy resistance [41,42]. Although endocrine therapy remains the backbone of ER^+^ management, primary resistance occurs in 15–20% of patients, and acquired resistance develops in approximately 30–40% of cases [40]. Importantly, loss of ER expression accounts for only a minority of resistant tumors; instead, persistent or even enhanced ER signaling often underlies therapeutic escape [43]. This distinction is critical: resistance in ER^+^ disease is not typically a loss-of-target phenomenon, but rather a reprogramming of receptor function and its downstream survival circuitry.

#### 2.3.3. Ligand-Independent ER Reactivation and Signaling Crosstalk

A central mechanism of endocrine escape involves ligand-independent activation of ERα through inflammatory and growth factor pathways. Enhanced interferon-α (IFNα)/JAK–STAT signaling has been shown to directly interact with ERα, promoting estrogen-independent transcriptional activation and sustaining survival gene expression in aromatase inhibitor (AI)-resistant cells [44]. STAT1 physically associates with ERα at estrogen response elements, reinforcing a transcriptional program that supports resistance and cell survival [45]. This inflammatory–hormonal crosstalk exemplifies a broader adaptive principle: resistant ER^+^ tumors maintain ER activity through alternative upstream inputs rather than eliminating ER dependency altogether. Collectively, these data indicate that resistant ER^+^ tumors preserve ER dependency while redirecting upstream regulatory inputs, thereby maintaining survival output under endocrine deprivation.

#### 2.3.4. Tumor Microenvironment-Driven ER Reprogramming

Beyond intrinsic tumor cell signaling, the tumor microenvironment (TME) plays a decisive role in ER modulation. Cancer-associated fibroblasts (CAFs) spatially coincide with reduced ERα expression and altered transcriptional output in luminal breast tumors [46]. CAFs selectively suppress canonical estrogen-responsive genes while preserving transcriptional programs associated with invasion, basal-like differentiation, and therapeutic resistance [47]. This selective ER reprogramming permits estrogen-independent growth while retaining survival pathways. Mechanistically, TGF-β and JAK signaling cascades were identified as mediators of CAF-induced ER modulation [48]. Thus, ER signaling plasticity reflects both intrinsic transcriptional rewiring and extrinsic stromal conditioning, establishing a permissive context for subsequent therapy resistance.

#### 2.3.5. Dormancy, EMP, and Late Recurrence

ER^+^ breast cancer uniquely exhibits a prolonged dormant phase at metastatic sites. In vivo intraductal xenograft models demonstrate that disseminated ER^+^ tumor cells proliferate more slowly than TNBC counterparts and display features of epithelial–mesenchymal plasticity (EMP) during dormancy [42]. Reduced CDH1 expression and increased ZEB1/2 expression characterize this dormant state [49]. Forced epithelial reconversion overcomes dormancy [50], indicating that EMP is not merely a metastatic trait but a functional regulator of latent survival. Complementary 3D culture systems modeling ER^+^ dormancy show that matrix composition and β1-integrin signaling influence long-term growth arrest and reactivation [41]. Autophagy-related pathways are enriched in dormant ER^+^ populations [51], and these cells are often enriched in stem-like features linking dormancy to cancer stem cell (CSC) biology [52]. Importantly, dormancy-associated plasticity programs may later confer tolerance to cytotoxic chemotherapy by preconditioning cells to survive oxidative, replicative, and metabolic stress.

#### 2.3.6. Transition from Endocrine Resistance to Chemotherapy Resistance

As ER^+^ disease progresses to metastatic stages, standard-of-care regimens incorporate CDK4/6 inhibitors and PI3K/AKT/mTOR pathway inhibitors in combination with endocrine therapy [53]. Resistance to these targeted agents frequently converges on shared adaptive programs: for example, these adaptive modules not only facilitate endocrine escape but also establish cross-resistance to cytotoxic chemotherapy by enhancing stress tolerance, metabolic buffering, and phenotypic plasticity [40]. Thus, chemotherapy resistance in ER^+^ breast cancer frequently represents the endpoint of a stepwise evolutionary process initiated by endocrine escape rather than an independent event.

### 2.4. HER2^+^ Breast Cancer: Targeted Therapy Failure and Cytotoxic Resistance

HER2^+^ breast cancer is characterized by amplification and/or overexpression of the ERBB2 gene, leading to constitutive activation of HER2-driven oncogenic signaling. HER2 forms homo- or heterodimers—preferentially with HER3—thereby activating downstream PI3K/AKT and MAPK cascades that promote proliferation, survival, angiogenesis, and metastatic competence [54,55]. Although anti-HER2-targeted therapies have dramatically improved survival, both primary and acquired resistance remain major clinical challenges [4].

#### 2.4.1. Molecular Basis of Anti-HER2 Resistance

Resistance arises through HER2 intratumoral heterogeneity (ITH), observed in up to 40% of cases [56] and associated with inferior response to trastuzumab and other anti-HER therapies [57]. Spatial heterogeneity permits survival of HER2-low subclones under therapeutic pressure [58]. Activation of downstream signaling independently of HER2 blockade also contributes to resistance. Dysregulation of the PI3K/AKT/mTOR axis through PIK3CA mutation or PTEN loss sustains survival signaling even during HER2 inhibition [29,30]. Persistent PI3K/AKT activation diminishes sensitivity to trastuzumab and lapatinib [59]. Extracellular matrix remodeling further modulates the response. Increased collagen density and alignment correlate with [3] poorer prognosis and reduced anti-HER2 responsiveness [60]. Collectively, HER2 resistance reflects both receptor-level heterogeneity and downstream pathway buffering that maintains survival signaling despite targeted inhibition.

#### 2.4.2. Failure of Targeted Therapy and Transition to Cytotoxic Resistance

Under sustained therapeutic pressure, clonal selection favors HER2-low/negative subclones and alternative receptor tyrosine kinase activation [4]. These adaptive changes not only impair targeted therapy efficacy but also contribute to broader cytotoxic resistance. Activation of PI3K/AKT signaling confers anti-apoptotic advantages that reduce sensitivity to taxanes and anthracyclines [61]. Residual cancer burden (RCB) after neoadjuvant therapy is strongly prognostic; RCB I patients exhibit worse survival compared to RCB 0 [62]. This suggests that minimal residual disease (MRD) reflects intrinsic or acquired resistance mechanisms that persist despite multimodal therapy. Residual disease therefore represents a biologically selected population enriched for stress-adaptive circuitry capable of withstanding both targeted and cytotoxic therapies.

### 2.5. Triple-Negative Breast Cancer (TNBC): Intrinsic and Acquired Chemoresistance

TNBC represents 10–15% of breast cancers and is characterized by high grade, early relapse [63], and reliance on cytotoxic chemotherapy [64].

#### 2.5.1. Molecular Heterogeneity as the Basis of Differential Chemosensitivity

TNBC comprises molecular subtypes with distinct vulnerabilities [65]. BL1 tumors demonstrate higher pCR rates to carboplatin-containing regimens, whereas LAR tumors exhibit significantly lower pCR rates [66]. These findings indicate that intrinsic chemoresistance in TNBC is subtype-dependent and closely linked to proliferative capacity and DNA damage response competency.

#### 2.5.2. Tumor Heterogeneity and Clonal Evolution Under Chemotherapeutic Pressure

Intratumoral heterogeneity drives acquired resistance through clonal selection [63]. Downregulation of MHC-I and epigenetic silencing of antigen-processing machinery impair CD8^+^ T-cell recognition [67]. Thus, chemoresistance in TNBC reflects both tumor-intrinsic adaptation and immune-mediated selection pressures.

#### 2.5.3. EMT, Plasticity and Stromal Remodeling

TGFβ/BMP signaling promotes EMT, stemness, and invasion [68]. ZL170 inhibition suppresses EMT and reduces stromal remodeling in TNBC models [69]. EMT-associated plasticity therefore establishes a stress-tolerant state that underlies both intrinsic and acquired chemoresistance.

#### 2.5.4. Immune Contexture and Limited Immunotherapy Benefit

The NeoTRIP Michelangelo trial showed no significant pCR improvement with atezolizumab addition [70], while PD-L1 expression remained a predictive factor [71]. Epigenetic suppression of HLA genes further limits immune infiltration [72]. Immune evasion mechanisms therefore cooperate with metabolic and plasticity programs to sustain TNBC chemoresistance.

#### 2.5.5. Metabolic Reprogramming and Microenvironmental Immunosuppression

Upregulated glutamine metabolism, fatty acid synthesis, and amino acid competition generate an immunosuppressive microenvironment [63]. LAR tumors demonstrate activated fatty acid networks driven by promoter hypomethylation, such as FASN [64]. Metabolic rewiring thus integrates with plasticity and immune suppression to reinforce chemotherapy tolerance.

## 3. Conserved Steroid Receptor-Driven Adaptive Circuits in Breast Cancer Chemoresistance

### 3.1. Redox Buffering and Ferroptosis Resistance Module

Cytotoxic agents—including anthracyclines, platinum compounds, and taxanes—promote reactive oxygen species (ROS) accumulation, lipid peroxidation, mitochondrial dysfunction, and iron-dependent oxidative injury. While these events can trigger apoptotic or ferroptotic cell death in sensitive tumors, resistant cancers frequently activate a coordinated redox defense network that suppresses ferroptotic execution and sustains survival under persistent oxidative stress [73,74]. Clinical and transcriptomic analyses indicate that enhanced antioxidant capacity, characterized by upregulation of NRF2, GPX4, and SOD1, is associated with a chemoresistant tumor phenotype [75,76]. These redox buffering mechanisms should not be interpreted as isolated antioxidant responses but rather as components of a coordinated adaptive tumor state integrating ferroptosis resistance, metabolic rewiring, and immune evasion [77]. These findings support the concept that enhanced antioxidant buffering capacity directly contributes to chemoresistance by neutralizing ROS and preventing lethal lipid peroxidation. In addition, NRF2 activation transcriptionally upregulates multiple antioxidant and detoxification genes—including SLC7A11 and enzymes involved in NADPH regeneration—thereby stabilizing redox homeostasis under genotoxic stress [78,79]. Ferroptosis sensitivity in breast cancer is tightly governed by the balance between ACSL4 and GPX4. ACSL4 enriches cellular membranes with polyunsaturated fatty acids (PUFAs), increasing susceptibility to peroxidation, whereas GPX4 detoxifies lipid hydroperoxides and prevents ferroptotic collapse. In patients receiving neoadjuvant chemotherapy, ACSL4 expression positively correlates with pathological complete response (pCR), whereas elevated GPX4 expression is associated with inferior outcomes [80]. These clinical data indicate that ferroptosis-competent tumors (high ACSL4/low GPX4) are more chemosensitive, whereas ferroptosis-resistant phenotypes preferentially survive cytotoxic stress. Importantly, steroid receptor signaling may influence this balance by modulating lipid metabolism and antioxidant gene expression programs, thereby indirectly altering the ferroptotic threshold [75,81]. Upstream of GSH metabolism, the cystine/glutamate antiporter SLC7A11 (xCT) regulates cystine import, sustaining intracellular GSH biosynthesis. Redox perturbations that downregulate SLC7A11 elevate intracellular ROS and can paradoxically induce compensatory activation of multidrug resistance pathways, including P-glycoprotein (P-gp)-mediated drug efflux [82]. Thus, SLC7A11 functions not only as a ferroptosis gatekeeper through GSH regulation but also as a molecular interface linking oxidative stress responses to canonical chemoresistance machinery. This dual role situates SLC7A11 at a critical intersection between redox control and drug transport adaptation [83]. Mitochondrial metabolism further reinforces this redox-adaptive circuit. Chemoresistant triple-negative breast cancer (TNBC) cells frequently display enhanced oxidative phosphorylation (OXPHOS) dependency rather than glycolytic predominance [79]. Increased OXPHOS activity elevates mitochondrial ROS production; however, resistant cells counterbalance this through coordinated upregulation of antioxidant enzymes, NADPH-generating pathways, and mitophagy programs. This establishes a high-flux redox equilibrium in which ROS production and detoxification are simultaneously elevated, preventing catastrophic lipid peroxidation while maintaining ATP supply and biosynthetic capacity [80,82]. Such redox recalibration permits tumor cells to tolerate sublethal oxidative stress that would otherwise induce ferroptosis. Importantly, ferroptosis resistance in breast cancer is not solely tumor-cell-intrinsic but also shaped by tumor microenvironmental interactions. In chemoresistant breast cancer, ferroptotic neutrophils suppress antitumor CD8^+^ T-cell activity via secretion of PGE2, IDO, and oxidized lipid mediators [83]. This immunosuppressive circuit is driven by IL1β^+^CXCL3^+^ CD4^+^ T cells that regulate MOAT1 expression and phospholipid remodeling in neutrophils, enhancing their ferroptotic susceptibility. Rather than promoting tumor eradication, immune cell ferroptosis can therefore remodel the microenvironment toward immune suppression and therapeutic resistance. These findings highlight the context dependency of ferroptosis: tumor cell ferroptosis promotes therapy response, whereas immune cell ferroptosis may facilitate resistance [84]. Collectively, these data define a conserved redox buffering–ferroptosis resistance module in steroid receptor-driven breast cancer in which cystine uptake (SLC7A11), GSH biosynthesis, GPX4-mediated lipid peroxide detoxification, ACSL4-dependent PUFA remodeling, mitochondrial OXPHOS-derived ROS flux, and tumor microenvironmental ferroptotic reprogramming operate as an integrated adaptive circuit. Rather than simply suppressing ROS, chemoresistant tumors recalibrate redox dynamics to maintain a controlled, sublethal oxidative state that promotes genomic instability tolerance, EMT-associated plasticity, and drug efflux activation while avoiding ferroptotic collapse. Therapeutically, disruption of this module may restore ferroptotic vulnerability. Strategies include GPX4 inhibition or ACSL4 activation to lower the ferroptotic threshold; targeting SLC7A11 to impair cystine import and GSH synthesis; inhibition of OXPHOS in metabolically rewired resistant tumors; and interference with IL1β-driven immune ferroptotic crosstalk within the tumor microenvironment. Importantly, these approaches are most likely to be effective when deployed in biomarker-selected tumors characterized by high NRF2/GPX4 expression, elevated OXPHOS signatures, or ferroptosis-resistant transcriptional profiles [74,75]. In sum, redox buffering represents a conserved adaptive circuit that mechanistically bridges steroid receptor signaling, metabolic reprogramming, immune remodeling, and multidrug resistance. Targeting this integrated network—rather than a single antioxidant node—may convert redox-adapted, ferroptosis-resistant tumors into ferroptosis-permissive and chemosensitive phenotypes.

### 3.2. DNA Damage Response (DDR) and Replication Stress Adaptation

Cytotoxic chemotherapy in breast cancer, including anthracyclines, platinum compounds, and taxanes, exerts antitumor effects primarily by inducing DNA double-strand breaks, interstrand crosslinks, replication fork collapse, and mitotic catastrophe. However, DNA damage response networks should not be viewed as passive repair systems. In chemoresistant contexts, DDR functions as a highly adaptable stress-buffering platform that enables tumor cells to tolerate genotoxic injury while preserving proliferative capacity under sustained therapeutic pressure. Increasing evidence suggests that in steroid receptor-positive and adaptive triple-negative breast cancers, DDR is reconfigured into a pro-survival framework that coordinates homologous recombination, replication fork stabilization, checkpoint activation, and transcriptional remodeling.

#### 3.2.1. Replication Stress as a Selective Pressure

Oncogenic signaling such as MYC amplification, cyclin E overexpression, and PI3K hyperactivation increases replication origin firing and nucleotide demand, thereby generating intrinsic replication stress even before chemotherapy exposure. Upon treatment with DNA-damaging agents, this pre-existing stress is amplified, resulting in single-stranded DNA accumulation, RPA recruitment, ATR activation, and progressive fork instability. Resistant breast cancer cells do not passively endure this stress. Instead, they actively remodel replication forks through coordinated ATR–CHK1 signaling and RAD51-dependent fork protection mechanisms [85,86,87,88]. Replication fork reversal and controlled processing are now understood as regulated survival responses rather than pathological byproducts of genomic instability [89]. Through fork remodeling, cancer cells slow replication progression, stabilize nascent DNA strands, and prevent irreversible fork collapse. In this setting, replication stress becomes a selective pressure that favors cells capable of reinforcing checkpoint signaling and maintaining fork integrity. Chemotherapy therefore does not simply eliminate tumor cells but selects for subpopulations that exhibit heightened replication stress tolerance.

#### 3.2.2. Homologous Recombination (HR) Reactivation and Rewiring

Homologous recombination deficiency, such as that caused by BRCA1 or BRCA2 loss, initially confers sensitivity to platinum agents and PARP inhibitors. However, acquired resistance frequently emerges through restoration of key HR functions. This restoration may occur through BRCA reversion mutations, re-establishment of RAD51 filament formation, loss of 53BP1–Shieldin pathway components, or replication fork protection mechanisms that operate independently of canonical HR repair [90]. These adaptive processes stabilize stalled forks and reduce double-strand break accumulation despite continued exposure to genotoxic therapy. Importantly, HR in resistant tumors does not necessarily revert to a fully wild-type state. Instead, selective reactivation of fork-protective modules may be sufficient to prevent catastrophic genomic fragmentation. HR components also participate in post-replicative gap filling and daughter-strand gap protection, thereby limiting replication-associated DNA collapse during persistent stress [85]. In this context, chemoresistance often reflects a functional rewiring of HR toward fork stabilization and damage tolerance rather than complete restoration of high-fidelity repair.

#### 3.2.3. ATR–CHK1 Axis and Checkpoint Dependency

The ATR–CHK1 signaling cascade orchestrates cellular adaptation to replication stress by suppressing late origin firing, stabilizing stalled forks, enforcing intra-S and G2/M checkpoint arrest, and coordinating recruitment of repair factors. Tumors characterized by chronic oncogene-driven replication stress become increasingly dependent on this pathway, a phenomenon often described as replication stress addiction [91]. Under continuous cytotoxic exposure, this checkpoint circuitry acts as a survival amplifier that allows cells to delay mitotic entry, resolve replication intermediates, and prevent mitotic catastrophe. Pharmacologic inhibition of ATR, CHK1, or WEE1 disrupts this buffering system and can precipitate replication catastrophe in highly stressed tumors [92]. These findings underscore that DDR signaling is not merely a defensive mechanism but a conditional dependency that sustains chemoresistant proliferation in the face of persistent DNA damage.

#### 3.2.4. DDR Plasticity and Epigenetic Modulation

Chemoresistant breast cancers frequently exhibit dynamic DDR rewiring mediated by transcriptional and epigenetic reprogramming. Alternative splicing of repair genes, alterations in post-translational modifications of key DDR proteins, epigenetic activation or silencing of homologous recombination components, and upregulation of translesion synthesis polymerases collectively contribute to damage tolerance [90,93]. These changes allow tumor cells to bypass platinum-induced crosslinks and replication-associated lesions while reducing acute chromosomal fragmentation. DNA damage tolerance mechanisms, particularly translesion synthesis polymerase activation, enable lesion bypass without immediate fork collapse. Although this strategy diminishes short-term cytotoxicity, it increases mutational burden and fuels tumor evolution. Thus, DDR plasticity simultaneously supports survival and accelerates genomic diversification, promoting long-term therapeutic resistance.

#### 3.2.5. Integration with Steroid Receptor-Driven Adaptive Circuits

Steroid receptor signaling intersects with DDR pathways at multiple regulatory layers. Estrogen receptor activity influences transcription of homologous recombination genes, including BRCA1 and RAD51, thereby modulating repair capacity in hormone receptor-positive breast cancer. Glucocorticoid receptor activation has been shown to enhance pro-survival signaling networks and reinforce checkpoint resilience under genotoxic stress. In certain breast cancer subsets, androgen receptor signaling can also influence DNA repair gene expression and chromatin accessibility at repair-associated loci.

### 3.3. Phenotypic Plasticity: EMT–Stemness–Lineage Switching

Phenotypic plasticity represents a central adaptive axis in breast cancer chemoresistance, enabling tumor cells to dynamically transition among epithelial, mesenchymal, stem-like, and lineage-shifted states in response to therapeutic stress. Rather than reflecting a binary epithelial-to-mesenchymal transition, accumulating evidence supports the existence of hybrid epithelial–mesenchymal states that retain partial epithelial adhesion while acquiring mesenchymal motility, metabolic flexibility, and stress tolerance [94,95]. These intermediate phenotypes exhibit enhanced metastatic competence, immune evasion, and drug tolerance and frequently serve as reservoirs for MRD and subsequent relapse. In breast cancer, EMT is tightly interwoven with cancer stem cell programs. Cells undergoing EMT often activate stemness-associated transcriptional networks involving SOX2, OCT4, NANOG, and ZEB1/2, thereby acquiring self-renewal capacity and resistance to cytotoxic stress [96]. However, stemness is not a fixed trait confined to a rare subpopulation. Non-stem cancer cells can reacquire stem-like features in response to inflammatory cytokines, hypoxia, oxidative stress, and chemotherapy-induced injury. This bidirectional interconversion underscores the concept of epithelial–mesenchymal plasticity, in which fluctuating transcriptional and epigenetic states generate intratumoral heterogeneity and facilitate adaptive resistance [94]. Plasticity therefore operates as a dynamic equilibrium rather than a linear progression. Steroid receptor signaling intersects with these plasticity programs in subtype-specific and context-dependent manners. In estrogen receptor-positive breast cancer, endocrine therapy can select for subpopulations with diminished epithelial differentiation and increased mesenchymal and stem-like features. Attenuation or loss of estrogen receptor signaling has been associated with upregulation of EMT transcription factors, including SNAIL, TWIST, and ZEB1, and with activation of TGF-β and Wnt/β-catenin pathways, thereby reinforcing mesenchymal reprogramming [97]. Conversely, persistent steroid receptor activity does not necessarily preserve epithelial identity. In certain contexts, estrogen receptor, progesterone receptor, or androgen receptor signaling can cooperate with growth factor pathways such as EGFR, HER2, and AXL to sustain hybrid EMT states that balance adhesion plasticity with survival signaling. These hybrid configurations may be particularly advantageous under therapeutic pressure because they preserve proliferative signaling while enabling adaptive motility and stress tolerance. Lineage switching adds an additional dimension to phenotypic adaptation. Under sustained chemotherapy or endocrine therapy, luminal breast cancer cells may undergo partial dedifferentiation toward basal-like or neuroendocrine-like phenotypes, accompanied by extensive chromatin remodeling and transcriptional rewiring. This process resembles developmental reprogramming events observed in aggressive malignancies in which mesenchymal and neural crest-like programs converge to enhance invasiveness and stress resilience [52,98]. Lineage infidelity weakens canonical receptor dependency and reduces sensitivity to receptor-targeted therapies, thereby expanding the spectrum of survival strategies available to tumor cells. Importantly, phenotypic plasticity is closely linked to dormancy and MRD biology. Disseminated tumor cells that have undergone partial EMT frequently adopt quiescent or slow-cycling states within distant microenvironmental niches, thereby evading immune surveillance and cytotoxic agents [96]. These cells maintain a poised chromatin landscape characterized by reversible epigenetic modifications and context-dependent transcriptional activation. Upon receiving permissive microenvironmental signals, dormant cells can rapidly re-enter the cell cycle and reconstitute heterogeneous tumor populations. EMT-associated plasticity therefore underpins not only dissemination but also long-term persistence and late metastatic relapse. At the mechanistic level, plasticity is stabilized through multilayered regulatory networks. Transcriptional feedback loops involving SNAIL or ZEB family members and the miR-200 axis maintain bistable or multistable states that permit reversible transitions. Epigenetic reprogramming, including histone modification dynamics and alterations in chromatin accessibility, reinforces state flexibility. Importantly, redox adaptation and metabolic rewiring contribute directly to the maintenance of mesenchymal and stem-like states. Elevated reactive oxygen species levels can activate EMT transcription factors and TGF-β signaling, while enhanced NADPH production and antioxidant buffering prevent oxidative catastrophe during mesenchymal transition. In parallel, replication stress and DNA damage response signaling influence transcriptional plasticity by modulating chromatin architecture and stress-responsive gene expression. Through these interactions, plasticity becomes integrated with the redox buffering and DDR modules described above, forming an interconnected adaptive landscape. Collectively, EMT–stemness–lineage switching should therefore be conceptualized as a dynamic continuum rather than discrete states. EMT-associated stemness and lineage reprogramming should therefore be conceptualized as a dynamic continuum in which steroid receptor signaling, redox balance, replication stress tolerance, and metabolic remodeling converge to shape cellular identity under therapeutic pressure [99,100,101]. In steroid receptor-driven breast cancer, these plasticity circuits enable transient disengagement from canonical receptor dependency, facilitate survival during genotoxic or endocrine stress, and subsequently permit re-expansion of heterogeneous tumor populations once selective pressure diminishes [52,102]. From a therapeutic perspective, durable control of chemoresistance requires disruption of the regulatory networks that stabilize plastic states rather than targeting a single differentiation marker [97,103]. Strategies that constrain EMT transcriptional circuits, inhibit AXL or TGF-β signaling, modulate chromatin remodeling enzymes, or interfere with redox-supported stemness programs have been shown to limit adaptive state transitions and reduce the emergence of drug-tolerant persister (DTP) populations [82,104]. Importantly, accumulating evidence suggests that multi-target phytotherapeutic compounds can concurrently modulate oxidative signaling, inflammatory pathways, and EMT transcriptional regulators, thereby destabilizing plastic equilibrium without requiring complete pathway blockade [73,105,106]. Recent evidence indicates that multi-target phytochemical formulations can coordinately regulate intracellular redox buffering, DNA damage signaling, and survival-associated kinase pathways rather than acting on a single oncogenic node [105,106]. For example, a combined herbal formulation derived from BK002 (*Achyranthes japonica* and *Melandrium firmum*) demonstrated ROS-dependent activation of CHOP, attenuation of PI3K activity, and induction of γH2AX-associated DNA damage markers, indicating functional coupling between oxidative perturbation and DDR modulation [29]. Further mechanistic profiling revealed suppression of DNMT1, Dicer, PD-L1, and PI3K/AKT signaling nodes through integrated transcriptomic and docking analyses, supporting a multi-node pharmacological targeting architecture [107]. Importantly, such coordinated modulation may reduce the adaptive redox–DDR tolerance threshold by concurrently weakening antioxidant buffering capacity and pro-survival kinase signaling [105,106]. Within the SR^3^ network framework proposed in this review, phytochemical interventions should therefore be conceptualized not as isolated antioxidant agents but as circuit-level destabilizers capable of perturbing stress-buffering modules across transcriptional, metabolic, and repair dimensions. In summary, phenotypic plasticity functions as a conserved adaptive module linking steroid receptor signaling to metastatic competence, therapy resistance, dormancy, and MRD persistence [94,108]. Recognition of the temporal and reversible nature of these state transitions, particularly at single-cell resolution, is increasingly viewed as essential for designing precision strategies aimed at preventing phenotypic escape and long-term relapse [109,110].

### 3.4. Metabolic Rewiring and NADPH Homeostasis

**Figure 1:**
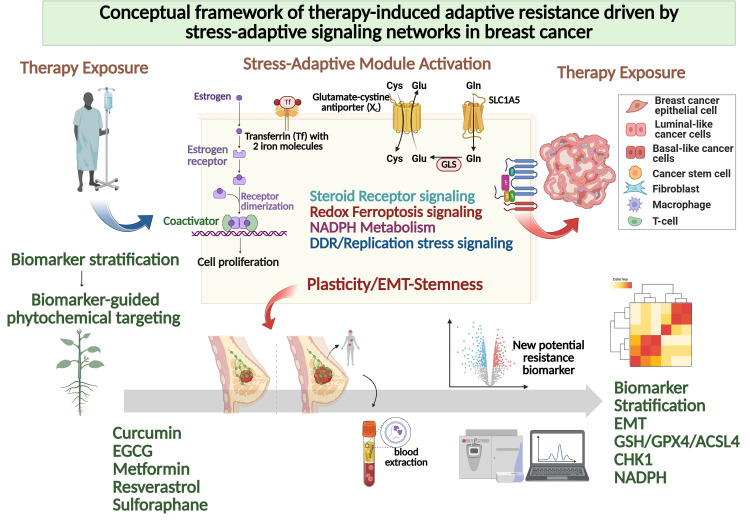
Systems-level SR^3^ network framework underlying stress-adaptive therapeutic resistance in breast cancer. This schematic illustrates the steroid receptor–redox–replication stress (SR^3^) network that integrates major stress-adaptive signaling modules driving therapeutic resistance in breast cancer. The framework highlights the functional coupling among redox–ferroptosis regulation, DDR–replication stress signaling, ISR–translation plasticity, and EMT–stemness programs. Metabolic buffering systems supporting NADPH homeostasis further stabilize redox adaptation. Bidirectional interactions between redox signaling and DDR pathways, as well as crosstalk with steroid receptor-dependent transcriptional programs, enable dynamic state transitions and adaptive plasticity that sustain therapy resistance. In addition, the schematic incorporates biomarker-guided stratification and downstream identification of potential resistance-associated biomarkers, illustrating the translational workflow from therapy exposure to adaptive state transitions and biomarker discovery. Abbreviations: DNA damage response (DDR); epithelial–mesenchymal transition (EMT); integrated stress response (ISR); steroid receptor–redox–replication stress network (SR^3^). Created in BioRender. Park, M.N. (2026) https://BioRender.com/b2bkmz4, accessed on 24 March 2026.

Metabolic rewiring in chemoresistant breast cancer is not merely a secondary consequence of proliferative demand. Rather, it represents a structured adaptive architecture that preserves redox stability, sustains biosynthetic flux, and maintains survival under persistent cytotoxic pressure. At the center of this architecture lies nicotinamide adenine dinucleotide phosphate in its reduced form, NADPH, which serves as the principal intracellular reducing currency coordinating antioxidant defense, lipid synthesis, nucleotide production, and replication competence. Resistant tumor cells maintain an elevated NADPH-to-NADP^+^ ratio to buffer reactive oxygen species while preserving redox-sensitive signaling cascades required for continued proliferation and plasticity [100,111,112]. Under endocrine therapy, platinum-based chemotherapy, or radiation exposure, oxidative pressure intensifies and selects for clones capable of reinforcing NADPH-generating pathways. NADPH is essential for regeneration of reduced glutathione via glutathione reductase and for maintenance of thioredoxin in its reduced state through thioredoxin reductase, thereby supporting detoxification of hydrogen peroxide and lipid peroxides [113]. Because glutathione peroxidase 4 depends on reduced glutathione to neutralize lipid hydroperoxides, sustained NADPH production directly contributes to ferroptosis resistance. In this framework, NADPH homeostasis does not simply counteract oxidative stress but actively stabilizes the ferroptotic threshold and enables survival in high-ROS environments. Multiple metabolic pathways converge to maintain NADPH availability. The oxidative branch of the pentose phosphate pathway, initiated by glucose-6-phosphate dehydrogenase, provides a dominant cytosolic source of reducing equivalents [114]. However, resistant breast cancer cells rarely depend on a single metabolic node. Cytosolic isocitrate dehydrogenase 1, mitochondrial isocitrate dehydrogenase 2, malic enzymes, and one-carbon metabolism pathways contribute additional NADPH pools, creating a distributed and compensatory network [105,111]. This redundancy confers metabolic robustness, ensuring that inhibition of a single pathway does not precipitate catastrophic redox imbalance. Steroid receptor signaling is functionally embedded within this reductive metabolic framework. Estrogen receptor, androgen receptor, and glucocorticoid receptor transcriptional programs influence enzymes within the pentose phosphate pathway, lipid biosynthesis, and mitochondrial oxidative metabolism, thereby indirectly sustaining NADPH flux [52,102]. Because NADPH is required for fatty acid elongation, desaturation, and cholesterol synthesis, enhanced reductive metabolism reinforces membrane remodeling and supports steroid biosynthesis. This establishes a reciprocal relationship in which steroid receptor signaling promotes NADPH production, while NADPH availability sustains anabolic processes that preserve receptor-driven tumor growth. In endocrine-resistant luminal tumors, this integration permits maintenance of anabolic and survival capacity despite pharmacologic inhibition of canonical receptor signaling pathways. Metabolic rewiring also underpins phenotypic plasticity. Proliferative epithelial states often rely on glycolytic intermediates and lipogenesis, whereas invasive or therapy-tolerant mesenchymal states may shift toward oxidative metabolism and exogenous lipid uptake. Such transitions require dynamic metabolic flexibility, allowing tumor cells to alternate substrate utilization in response to microenvironmental constraints [108,115]. This flexibility supports maintenance of mitochondrial redox balance and ensures continued NADPH regeneration during state transitions. Elevated NADPH further stabilizes mesenchymal and stem-like programs by buffering oxidative stress that would otherwise trigger apoptotic or ferroptotic collapse during epithelial–mesenchymal reprogramming. Importantly, NADPH availability also intersects with DNA damage response resilience. Reductive power supports ribonucleotide reductase activity, nucleotide biosynthesis, and replication fork progression, thereby linking metabolic rewiring to replication stress tolerance. Adequate NADPH levels limit excessive oxidative DNA damage and facilitate repair pathway activity, allowing cells to endure sublethal genotoxic injury without activating irreversible apoptosis [85,101]. In this context, metabolic adaptation directly reinforces DDR plasticity and sustains proliferative recovery following chemotherapy exposure. Collectively, metabolic rewiring in breast cancer chemoresistance should be viewed as a coordinated NADPH-centered adaptive network that integrates redox buffering, ferroptosis resistance, steroid receptor signaling, replication stress tolerance, and phenotypic plasticity. Rather than functioning as isolated metabolic adjustments, these pathways operate as interconnected modules that stabilize tumor survival under therapeutic stress. Targeting components of this reductive network, including glucose-6-phosphate dehydrogenase, isocitrate dehydrogenases, malic enzymes, or downstream antioxidant systems, may destabilize redox equilibrium and sensitize resistant tumors to oxidative and genotoxic therapies. Importantly, several phytotherapeutic compounds have been reported to modulate pentose phosphate pathway flux, inhibit NADPH-producing enzymes, attenuate NRF2 signaling, or disrupt lipid biosynthetic pathways [73,105,106]. By simultaneously affecting multiple nodes within the NADPH-centered network, such multi-target interventions may reduce metabolic redundancy and lower the adaptive threshold required for chemoresistant survival. In this light, metabolic vulnerability in breast cancer may not reside in a single enzyme but in the coordinated reductive buffering architecture that sustains therapeutic tolerance. The core stress-adaptive modules sustaining therapeutic resistance in breast cancer are summarized in Table 1 and Table 2, which define the mechanistic and pharmacological landscape of redox-driven adaptive programs and their modulation by plant-derived compounds. In particular, Table 1 provides a state-oriented framework linking redox–EMT phenotypes with ferroptosis resistance, metabolic adaptation, and clinically relevant biomarker readouts, while Table 2 outlines candidate phytochemical modulators targeting these stress-adaptive pathways. To conceptually integrate these interconnected adaptive programs, Figure 1 illustrates the steroid receptor–redox–replication stress (SR^3^) network, highlighting the systems-level coupling among redox–ferroptosis regulation, DNA damage response (DDR)–replication stress, integrated stress response (ISR)–translation plasticity, and EMT–stemness programs that collectively sustain therapeutic resistance in breast cancer. Importantly, this integrated framework can be extended toward a biomarker-guided clinical trial architecture in which the state-based features summarized in Table 1 inform patient stratification and therapeutic allocation, thereby linking mechanistic classification with translational decision-making strategies [77].

**Table 1:** Core adaptive stress modules underlying therapeutic resistance in breast cancer.

Adaptive Module	Key Molecular Nodes	In Vitro Evidence	Evidence Type
Redox–ferroptosis buffering	SLC7A11, GPX4, NRF2–KEAP1, lipid peroxide buffering	Metformin disrupts SLC7A11-dependent redox buffering; Resveratrol destabilizes GPX4-mediated lipid peroxide control via NEDD4L [112]; EGCG enhances paclitaxel sensitivity via redox modulation [116,117]; Curcumin and berberine synergistically perturb redox–survival signaling networks [113]	Preclinical (in vitro/in vivo)
DDR/replication-stress adaptation	ATR–CHK1 axis; RAD51; checkpoint reinforcement	Genistein exhibits multi-target signaling effects with DDR-adjacent modulation	Preclinical; pathway-supportive modulation
EMT–plasticity Axis	Wnt–β-catenin; AXL; TGF-β; CSC transcriptional regulators	Sulforaphane suppresses breast CSC self-renewal and Wnt–β-catenin signaling [118,119]; additional EMT-modulatory phytochemicals	Preclinical
NADPH-centered metabolic robustness	PPP/G6PD, IDH1/2, ME1/3, one-carbon; NADPH–GSH/TRX coupling; OXPHOS vs. glycolysis switching	Catechin gallates inhibit G6PD [120]; EGCG suppresses glucose metabolism in BC models [121]; Berberine induces ROS–mitochondrial apoptosis [122]	Preclinical metabolic targeting

Abbreviations: AXL (AXL receptor tyrosine kinase); ATR (ataxia telangiectasia and Rad3-related); CHK1 (checkpoint kinase 1); CSC (cancer stem cell); DDR (DNA damage response); EGCG (epigallocatechin gallate); G6PD (glucose-6-phosphate dehydrogenase); GPX4 (glutathione peroxidase 4); GSH (glutathione); IDH1/2 (isocitrate dehydrogenase 1/2); KEAP1 (Kelch-like ECH-associated protein 1); ME1/3 (malic enzyme 1/3); NADPH (nicotinamide adenine dinucleotide phosphate); NEDD4L (neural precursor cell expressed developmentally downregulated 4-like); NRF2 (nuclear factor erythroid 2-related factor 2); OXPHOS (oxidative phosphorylation); PPP (pentose phosphate pathway); RAD51 (RAD51 recombinase); SLC7A11 (solute carrier family 7 member 11); TGF-β (transforming growth factor beta); TRX (thioredoxin).

**Table 2:** Metabolic robustness and pharmacological modulators targeting adaptive stress modules.

Adaptive Module	Key Molecular Nodes	In Vitro Evidence	In Vivo Evidence	Clinical Correlation
Redox–ferroptosis buffering	SLC7A11 (xCT), GSH, GPX4, ACSL4, NRF2–KEAP1; lipid–ROS threshold regulation	Therapy-triggered ROS and lipid peroxidation are buffered via ↑ GSH, ↑ GPX4, ↑ NRF2 signaling; ACSL4-high phenotypes show ferroptosis permissiveness; xCT integrates redox tone with MDR features [75,79,80,82,83,84]	Resistant TNBC exhibits oxidative metabolism-linked redox adaptation; TME-associated ferroptosis programs may become immunosuppressive [80,82]	High NRF2/GPX4/SOD antioxidant signatures are associated with poor chemotherapy response and relapse; ACSL4–GPX4 balance trends correlate with pCR outcomes [79,83,84]
DDR/replication-stress adaptation	ATR–CHK1–WEE1 checkpoint axis; RAD51-mediated fork protection; BRCA1/2; 53BP1/Shieldin; TLS/DDT tolerance	Chronic replication stress induces checkpoint addiction; fork stabilization and HR modules sustain survival under platinum/anthracycline pressure [85,89,123,124]	ATR/CHK1/WEE1-targeted combinations demonstrate activity in resistant models; fork protection phenotypes track with resistance evolution [90,91,125]	HRD confers initial sensitivity; resistance emerges via HR restoration and fork protection; “replication-stress–high” tumors rationalize ATR/CHK1/WEE1-based combinations [90,91,125]
EMT–stemness–lineage/state switching	ZEB1/2, SNAIL, TWIST, SOX2/OCT4/NANOG; AXL; TGF-β; Wnt–β-catenin; MRD/dormancy programs	Hybrid E/M states expand under therapy; CSC-like (CD44/ALDH) enrichment; lineage infidelity under therapeutic pressure [94,99,126,127]	MRD-like slow-cycling populations seed relapse and metastasis; EMT/AXL/TGF-β targeting constrains state transitions in vivo [52,96,97]	EMT/CSC signatures correlate with relapse risk; state-transition dynamics explain relapse despite target suppression” [52,96,97]

Abbreviations: ↑ Upregulation; ACSL4 (Acyl-CoA synthetase long-chain family member 4); ALDH (aldehyde dehydrogenase); β-catenin (Beta-catenin); BRCA1/2 (Breast cancer susceptibility gene 1/2); CD44 (Cluster of differentiation 44); CHK1 (checkpoint kinase 1); CSC (cancer stem cell); DDT (DNA damage tolerance); DDR (DNA damage response); EMT (epithelial–mesenchymal transition); GPX4 (glutathione peroxidase 4); GSH (glutathione); HR (homologous recombination); HRD (homologous recombination deficiency); KEAP1 (Kelch-like ECH-associated protein 1); MDR (multidrug resistance); MRD (minimal residual disease); NRF2 (Nuclear factor erythroid 2–related factor 2); RAD51 (RAD51 recombinase); SLC7A11 (Solute carrier family 7 member 11); SNAIL (Snail family transcriptional repressor 1); SOX2 (SRY-box transcription factor 2); TLS (translesion synthesis); TGF-β (Transforming growth factor beta); TNBC (triple-negative breast cancer); TWIST (Twist family bHLH transcription factor 1); WEE1 (WEE1 G2 checkpoint kinase); Wnt (Wingless/Integrated signaling pathway); xCT (cystine/glutamate antiporter; encoded by SLC7A11); ZEB1/2 (Zinc finger E-box binding homeobox 1/2).

### 3.5. Integrative Paragraph

Taken together, the redox buffering module, replication stress adaptation, phenotypic plasticity, and NADPH-centered metabolic rewiring should not be viewed as isolated resistance mechanisms. Instead, these processes operate as interdependent adaptive layers that collectively stabilize breast cancer survival under sustained therapeutic pressure [100,101]. Enhanced antioxidant capacity preserves replication competence; replication stress signaling influences transcriptional plasticity; metabolic NADPH homeostasis sustains both redox equilibrium and DNA repair efficiency; and phenotypic state transitions enable transient disengagement from canonical receptor dependency while maintaining stress tolerance. Through this coordinated integration, steroid receptor-driven breast cancers acquire a flexible yet robust survival architecture that allows dynamic adjustment to endocrine, genotoxic, and oxidative therapies [52,102]. Importantly, this adaptive integration helps explain why targeting a single pathway rarely produces durable responses. Disruption of one node within the network is often buffered by compensatory activation of parallel modules, reflecting the intrinsic plasticity of resistant tumors. Understanding chemoresistance therefore requires a systems-level appreciation of how redox control, DNA repair resilience, metabolic flux, and cellular identity co-evolve under treatment pressure. Such an integrative perspective provides a conceptual foundation for designing multi-target therapeutic strategies capable of destabilizing adaptive equilibrium and preventing long-term relapse. Within the SR^3^ framework, tumor microenvironmental components—including stromal and immune elements—should be interpreted as modulatory layers that reinforce and stabilize adaptive stress-response circuits rather than as primary circuit drivers. Within this framework, redox buffering and ISR modules predominantly function as feedback-regulated circuits; DDR signaling retains features of both linear and adaptive checkpoint pathways; and phenotypic plasticity, together with epigenetic reprogramming, primarily contributes to persistent state stabilization.

## 4. Why Resistance-Overcoming Strategies Repeatedly Fail

### 4.1. Intratumoral Heterogeneity and State Transition

Intratumoral heterogeneity represents a structural limitation to durable therapeutic response, not simply because tumors harbor genetically distinct subclones, but because malignant populations continuously redistribute across multiple interconvertible phenotypic states. Single-cell transcriptomic analyses have demonstrated that within a single tumor mass, cancer cells populate a structured spectrum of transcriptional states that frequently mirror developmental hierarchies of the tissue of origin [110]. These states are not randomly assembled. Rather, they are constrained by lineage-specific transcriptional circuits and epigenetic architectures that define the boundaries of accessible cellular identity. Epigenetic heterogeneity is central to sustaining this diversity. Unlike genetic mutations, epigenetic modifications are reversible and environmentally responsive, allowing rapid phenotypic adaptation without the requirement for new clonal selection events [128]. Fluctuations in DNA methylation, chromatin accessibility, and histone modifications generate a non-genetic dispersion of transcriptional programs across tumor cell populations. This variability permits transient activation of stress-responsive, mesenchymal-like, interferon-inducible, or stemness-associated modules that may exist at low frequency under baseline conditions but expand under therapeutic stress. In this context, heterogeneity is not a static property of tumor architecture but a dynamic reservoir of potential cellular states. Importantly, these states are connected through bidirectional transitions rather than fixed differentiation hierarchies. In small cell lung carcinoma, single-cell analyses have revealed commutable transcriptional states governed by dynamic equilibrium rather than unidirectional lineage commitment [106,129]. Such equilibrium behavior implies that eliminating a dominant state does not eradicate the tumor. Instead, residual populations can repopulate depleted phenotypes through plastic transitions that remain permissible within the tumor’s epigenetic landscape. Resistance therefore emerges not solely from survival of resistant clones but from redistribution within a constrained state-space [108]. Accordingly, the relevant therapeutic object is not only the abundance of a given resistant population but also the topology of the state landscape and the permissiveness of the transitions that connect stress-tolerant states to proliferative re-growth states [110]. Within this framework, therapeutic interventions that selectively eliminate proliferative or lineage-committed cells often perturb the state distribution without collapsing the underlying landscape. Slow-cycling, stress-adapted, or mesenchymal-like states may transiently dominate under treatment pressure and subsequently re-expand into proliferative phenotypes once selective constraints shift. Resistance-overcoming strategies thus frequently fail because they target a node within the state network while leaving intact the transitions that regenerate it. Intratumoral heterogeneity should therefore be interpreted not merely as clonal genetic diversity but as a dynamic state-space problem. Without constraining the accessibility of alternative phenotypic states or destabilizing the kinetics of state transitions, therapeutic suppression of a dominant pathway is unlikely to yield durable eradication. Resistance, in this sense, represents a predictable systems-level outcome of a plastic and self-replenishing tumor architecture. Within breast cancer, this framework is particularly relevant because endocrine pressure, HER2 blockade, and cytotoxic stress each reshape state distributions in subtype-specific ways, while preserving a shared capacity for reversible transitions into DTP states that later reseed proliferative outgrowth.

### 4.2. Treatment-Induced State Reprogramming

Resistance-overcoming strategies fail not only because resistant populations pre-exist but because therapy itself actively reshapes the tumor state landscape. Cytotoxic agents, targeted inhibitors, endocrine therapies, and metabolic interventions function as selective pressures that induce transcriptional and epigenetic remodeling, thereby generating new therapy-tolerant phenotypes. Treatment therefore acts not merely as a filter but as a state-inducing perturbation. Conceptually, this distinction matters because a purely selective model predicts that intensifying target inhibition should progressively reduce resistance, whereas a state-inducing model predicts that stress intensity, scheduling, and microenvironmental context can actively increase the accessibility of tolerant states even when initial cytoreduction is achieved.

#### 4.2.1. Chemotherapy as a State-Inducing Stress Signal

Single-cell time-series analysis of triple-negative breast cancer under platinum exposure demonstrated that drug treatment induces dynamic transcriptomic reprogramming rather than uniform clonal selection [110]. High-fitness copy-number-driven clones may undergo clonal expansion, yet low-fitness populations exhibit substantial transcriptional plasticity during exposure and withdrawal phases. Pseudotime reconstruction revealed hysteresis, indicating that post-treatment cells do not fully revert to their original pre-treatment configuration but instead stabilize within newly formed intermediate transcriptional states [130]. This observation carries important conceptual implications. Therapy does not simply enrich pre-existing resistant clones. It perturbs the regulatory network of the tumor, shifting cells toward alternative attractor states that were previously inaccessible or only weakly populated. These therapy-induced states may exhibit enhanced stress tolerance, altered metabolic dependencies, modified receptor signaling hierarchies, or epigenetically reinforced survival programs. Once stabilized, such states can persist even after drug withdrawal, creating a memory of treatment exposure embedded within the tumor’s transcriptional architecture. In this light, therapeutic intervention can increase phenotypic diversity by expanding the occupancy of intermediate or hybrid states that are competent for survival under stress yet remain capable of re-entry into proliferative programs. By inducing new stable or semi-stable phenotypes within the tumor state-space, treatment may inadvertently expand the adaptive repertoire available to malignant cells. Resistance-overcoming strategies therefore fail not simply because they are insufficiently potent but because they do not account for therapy-driven reprogramming that reshapes the landscape they aim to suppress.

#### 4.2.2. Translational Reprogramming and the Integrated Stress Response

Hypoxia, mTOR inhibition, and cytotoxic chemotherapy converge on a common adaptive pathway by inducing phosphorylation of eIF2α and activation of the integrated stress response [131]. These transcripts encode regulators of stemness and epithelial–mesenchymal transition, thereby promoting acquisition of stress-tolerant and stem cell-like phenotypes. Importantly, mTOR inhibition or chemotherapy recapitulates aspects of hypoxia-induced translational control, indicating that distinct therapeutic stresses converge mechanistically on ISR-dependent translational reprogramming. In this context, therapy does not simply reduce protein synthesis but reshapes the proteomic landscape toward plasticity-promoting factors. Experimental blockade of ISR signaling using pharmacologic agents such as ISRIB attenuates therapy-induced stemness and limits emergence of drug-tolerant states [131,132]. These findings suggest that stress-adaptive translational circuits are not passive consequences of therapy but active drivers of phenotypic reprogramming. Thus, therapeutic stress reshapes cell identity at the level of translation, reinforcing plasticity rather than merely selecting for pre-existing resistant clones. Within the logic of this review, ISR is therefore positioned as a mechanistic bridge between chemotherapy-induced stress and the emergence of plastic, therapy-tolerant states that later feed into recurrent disease.

#### 4.2.3. JAK–STAT-Driven Mesenchymal Transition

Inflammatory signaling provides another axis through which therapy reshapes tumor state architecture. In inflammatory breast cancer, chemotherapy-resistant derivatives display enrichment of inflammatory and EMT-associated transcriptional programs driven by JAK2–STAT3 signaling. Single-cell analyses demonstrate that chemotherapy promotes a shift from luminal toward basal and mesenchymal transcriptional states, accompanied by expansion of CD44-positive and phosphorylated STAT3-positive subpopulations [133]. Concurrent activation of cAMP and PKA signaling further stabilizes CREB-dependent transcriptional programs associated with stress adaptation. These observations indicate that treatment-induced inflammation is not an epiphenomenon but a mechanistic requirement for stabilization of mesenchymal chemoresistant states. Pharmacologic inhibition of JAK2–STAT3 signaling in combination with chemotherapy prevents expansion of mesenchymal-like populations and constrains resistant-state formation [134]. In this setting, inflammation functions as a state-stabilizing force that converts transient stress responses into durable transcriptional identities. Resistance therefore emerges from inflammatory reinforcement of plastic transitions rather than from simple survival of resistant clones. These data also highlight why suppression of tumor-intrinsic oncogenic signaling alone can be insufficient, because cytokine-driven reinforcement can stabilize resistant identity even when initial cytoreduction is achieved.

#### 4.2.4. Metabolic Rewiring Under Therapeutic Pressure

Metabolic plasticity represents a key dimension of therapy-induced state adaptation, with breast cancer cells reconfiguring metabolic flux between glycolysis, oxidative phosphorylation, and alternative substrates under cytotoxic stress. This reconfiguration frequently increases NADPH production, enhances reactive oxygen species buffering capacity, and stabilizes antioxidant responses that protect against lethal oxidative injury [135,136]. Crucially, metabolic states are not fixed. Cells exposed to metabolic inhibitors often compensate by redistributing flux through parallel NADPH-generating pathways or by increasing reliance on mitochondrial oxidative metabolism. As a result, strategies targeting a single metabolic node frequently fail because tumor cells do not remain metabolically static. Instead, they dynamically reorganize reductive and oxidative pathways to maintain redox balance and survival. Therapeutic pressure therefore induces a metabolic reprogramming trajectory that expands adaptive capacity rather than constraining it. Consistent with Section 3, this metabolic rewiring is best interpreted as a circuit-support layer that sustains ISR output and plasticity programs by maintaining redox permissiveness under continuous stress.

#### 4.2.5. Stromal Reprogramming and Immune Context

Resistance is not exclusively tumor-intrinsic. Therapeutic stress also reprograms the tumor microenvironment, reshaping immune composition and stromal signaling networks. In metastatic breast cancer models, stromal p38 MAPKα signaling modifies immune cell recruitment, macrophage polarization, and interferon gamma-dependent immune circuits, thereby creating an immunosuppressive niche that supports tumor persistence [137]. Inhibition of stromal p38 signaling sensitizes tumors to immunotherapy, underscoring the role of tumor–stroma co-adaptation in therapeutic failure. These findings indicate that treatment acts not only on malignant cells but also on the surrounding stromal and immune compartments. Stress-induced remodeling of the microenvironment can stabilize residual tumor populations and facilitate re-expansion following initial therapeutic response. Resistance therefore arises from coordinated reprogramming across tumor and stromal compartments rather than from isolated tumor cell survival.

#### 4.2.6. Therapy as an Evolutionary State-Generator

Therapy functions as an evolutionary state-generator rather than a purely selective filter [109,138]. While genotypically advantageous clones may expand under treatment, accumulating single-cell and longitudinal analyses demonstrate that therapeutic stress simultaneously induces transcriptional, translational, metabolic, and microenvironmental reprogramming that generates new adaptive states within the tumor ecosystem [115,139,140]. Through activation of the integrated stress response, inflammatory reinforcement via JAK–STAT signaling, redistribution of metabolic fluxes supporting NADPH homeostasis, and stromal remodeling, treatment reshapes the regulatory landscape that defines cellular identity and phenotypic accessibility [104,131,133]. Resistance-overcoming strategies repeatedly fail when they target only one dimension of this adaptive architecture. Experimental and clinical observations indicate that suppression of a dominant oncogenic pathway can eliminate a proliferative phenotype yet leave intact the epigenetically permissive transitions that regenerate it [109,141]. In the absence of interventions that constrain state accessibility or destabilize transition kinetics across interconnected stress-response, metabolic, and microenvironmental axes, therapeutic pressure continues to generate alternative attractor states capable of sustaining tumor survival [115,138]. Resistance is therefore more accurately understood as a dynamic systems-level response driven by stress signaling, epigenetic plasticity, metabolic buffering, and tumor–stroma co-evolution rather than as a fixed clonal attribute [133,142]. Framed this way, the practical implication is that durable response requires circuit-level containment that simultaneously limits state-transition permissiveness and disrupts the buffering programs that allow persister survival during stress exposure.

#### 4.2.7. Clinical Correlates of Persistent Therapy-Induced States (Metastatic Memory)

Although the concept of therapy-induced state persistence has been extensively characterized in preclinical models, converging clinical evidence supports the existence of residual tumor cell populations that retain adaptive survival programs following treatment. MRD, defined as occult tumor burden undetectable by conventional imaging, has emerged as a clinically measurable correlate of these persistent states and is strongly associated with recurrence and metastasis in breast cancer patients [143]. Advances in liquid biopsy technologies, including circulating tumor cells (CTCs), circulating tumor DNA (ctDNA), and exosome-based profiling, enable dynamic monitoring of MRD and provide an early indication of disease relapse prior to overt clinical progression [144]. Beyond MRD detection, longitudinal analyses of circulating tumor cells reveal that both CTC burden and the presence of CTC clusters are significantly associated with inferior progression-free and overall survival (OS) in metastatic breast cancer, indicating that persistent circulating tumor populations reflect clinically relevant adaptive survival states [145]. Importantly, these circulating populations are not static; rather, they exhibit dynamic changes during treatment, suggesting that therapy not only selects but actively reshapes tumor cell phenotypic states over time. Mechanistically, emerging clinical and translational evidence further supports the persistence of therapy-tolerant cell populations that survive initial treatment without immediate acquisition of stable genetic resistance. DTP cells and dormant tumor cell populations have been implicated as reservoirs for late relapse, maintaining viability through non-genetic adaptive mechanisms, including transcriptional plasticity, metabolic rewiring, and microenvironmental support [146]. These persistent cell states often overlap with minimal residual disease and may remain clinically undetectable for extended periods before reactivation and metastatic outgrowth. In parallel, therapy-induced senescent cell populations contribute to a persistent tumor-supportive microenvironment that can facilitate immune evasion, chronic inflammation, and eventual tumor recurrence. Accumulation of senescent cells within the tumor microenvironment has been shown to promote long-term treatment resistance and may represent an additional layer of state persistence beyond tumor-intrinsic mechanisms [147]. Collectively, these clinical observations provide a measurable and translationally relevant foundation for the concept of “metastatic memory,” in which prior therapeutic exposure imprints durable adaptive states within tumor cell populations and their microenvironment. In this framework, metastatic recurrence is not solely the consequence of clonal genetic evolution but reflects the persistence and reactivation of therapy-induced phenotypic states that remain buffered within the tumor ecosystem. Accordingly, metastatic memory should be interpreted as a clinically observable, hypothesis-generating construct that links MRD, persister cell biology, and microenvironmental remodeling into a unified model of long-term therapeutic resistance. Importantly, these observations suggest that metastatic progression may arise not only from de novo genetic evolution but also from reactivation of pre-existing adaptive circuit states established during prior therapy exposure. Recent evidence from metastatic biology reviews further supports the role of redox imbalance and metabolic plasticity as key determinants of metastatic organotropism and tumor survival during dissemination, providing a clinical–translational context for persistent adaptive states underlying metastatic memory [148].

## 5. Circuit-Guided Combination Strategy in Breast Cancer

### 5.1. Targeting Redox Buffering + Plasticity Simultaneously

Breast cancer progression and therapeutic resistance are not driven by isolated oncogenic pathways but by an adaptive biological circuitry that integrates redox homeostasis, phenotypic plasticity, metabolic rewiring, and DNA damage response signaling. Accumulating evidence indicates that monotherapeutic targeting of individual signaling axes yields only transient responses because tumor cells compensate through parallel circuit reactivation. Combination approaches in triple-negative breast cancer have illustrated that inhibition of single nodes such as PI3K, EGFR, or MEK rarely produces durable control due to compensatory activation of alternative survival programs [149]. These findings suggest that resistance emerges at the systems level rather than at the level of a single molecular target. Persistent oxidative stress is a defining feature of aggressive breast cancer. Oncogenic signaling, mitochondrial dysfunction, and metabolic acceleration generate continuous reactive oxygen species burden. To survive under these conditions, tumor cells reinforce antioxidant buffering systems. Central to this buffering capacity are the SLC7A11-driven cystine import pathway, glutathione synthesis, and GPX4-mediated detoxification of lipid peroxides. In parallel, chronic oxidative DNA lesions activate base excision repair and PARP-dependent repair pathways, further strengthening cellular resilience against redox-induced genomic instability [150]. DNA damage response signaling therefore functions not only as a genome maintenance mechanism but also as an adaptive redox stabilizer in breast cancer [151]. The EMAT-based stratification framework demonstrates that metastatic propensity follows a continuum of plastic cell states characterized by variable stemness and invasiveness [152]. These plastic states are tightly linked to redox adaptability. In addition, emerging evidence indicates that redox buffering systems are closely integrated with growth factor receptor signaling networks that drive therapeutic resistance. The NRF2 pathway functions as a master regulator of antioxidant defense and promotes survival of therapy-resistant cancer cells by upregulating detoxification enzymes and ferroptosis-protective systems such as GPX4. Persistent activation of NRF2 has been observed in resistant tumor populations and is associated with increased invasive capacity, epithelial–mesenchymal transition, and metabolic adaptation [153]. Notably, redox signaling also intersects with receptor tyrosine kinase pathways that govern breast cancer progression. Members of the HER family, particularly HER2 and EGFR, activate downstream PI3K–AKT and MAPK signaling cascades that reinforce proliferative and survival programs. NRF2-mediated antioxidant signaling can cooperate with HER-family receptor pathways to enhance tumor cell survival, maintain cancer stem cell populations, and promote resistance to chemotherapy and targeted therapy [154]. Cancer stem cell populations characterized by elevated ALDH activity frequently exhibit high NRF2 signaling and reduced intracellular ROS levels, enabling these cells to tolerate oxidative stress induced by chemotherapy. This redox-protected stem-like state contributes to tumor recurrence and metastatic dissemination by allowing resistant subpopulations to persist under therapeutic pressure [154,155]. Cells occupying highly plastic or stem-like states display enhanced antioxidant capacity and increased tolerance to oxidative stress. Reactive oxygen species can promote EMT transcriptional programs through activation of redox-sensitive signaling networks, whereas mesenchymal states reinforce ferroptosis resistance by upregulating glutathione-dependent pathways [156]. Targeting only one component of this circuitry has repeatedly proven insufficient. PARP inhibitors demonstrate significant benefit in homologous recombination-deficient populations [150], yet resistance develops through restoration of repair capacity or metabolic compensation. Similarly, targeted inhibition of growth factor pathways in TNBC often fails to prevent phenotypic switching and adaptive survival [149]. These patterns underscore that adaptive buffering systems must be disrupted simultaneously to prevent circuit-level compensation. A circuit-guided strategy therefore requires coordinated destabilization of antioxidant buffering together with restriction of phenotypic plasticity. Suppression of SLC7A11 or GPX4 activity increases vulnerability to lipid peroxidation and ferroptotic stress. However, unless plasticity signaling pathways such as STAT3 or other EMT-associated networks are concurrently restrained, tumor cells may transition toward alternative survival states. Integrating redox destabilization with modulation of plasticity-associated transcriptional programs and DDR signaling provides a mechanistic framework for collapsing adaptive loops rather than merely inhibiting a single effector. Clinically, patients whose tumors exhibit elevated SLC7A11 expression, enhanced GPX4 activity, high-EMAT signatures, or increased PARP dependency may represent biologically defined subgroups in which redox buffering and plasticity circuits are dominant [150,152]. In such contexts, rational combination strategies designed to simultaneously impair antioxidant defenses and constrain phenotypic flexibility may prevent state switching and limit metastatic progression. In summary, breast cancer therapeutic resistance should be conceptualized as an emergent property of interconnected redox, plasticity, and DNA repair networks. Effective combination therapy must therefore be designed at the circuit level, aiming to dismantle adaptive buffering systems and suppress phenotypic transitions that enable survival under therapeutic stress.

### 5.2. Metabolic–ISR Dual Interference

Breast cancer cells, particularly in triple-negative breast cancer, survive under metabolic stress by simultaneously rewiring NADPH production and activating the integrated stress response. These two adaptive systems are not independent. Rather, they function as a coordinated buffering circuit that stabilizes redox homeostasis and sustains cellular plasticity under therapeutic pressure. NADPH serves as the principal reducing currency required for glutathione regeneration and lipid peroxide detoxification. Through activation of the pentose phosphate pathway, malic enzyme activity, and mitochondrial one-carbon metabolism, cancer cells maintain a continuous NADPH flux that supports GPX4 activity and suppresses ferroptotic lipid damage. However, metabolic stressors such as glucose deprivation, mitochondrial dysfunction, or oxidative overload disrupt this reductive supply. Under these conditions, cells activate eIF2α phosphorylation and induce ATF4 translation as a compensatory survival response. Evidence from radioresistant TNBC models demonstrates that persistent eIF2α phosphorylation enhances ATF4-dependent transcription of SLC7A11 and glutathione biosynthetic enzymes, leading to increased intracellular GSH pools and improved ROS detoxification capacity [157,158]. This ISR-driven antioxidant reinforcement effectively couples translational control to metabolic redox buffering. In parallel, ISR activation under glucose depletion induces mitochondrial SLC1A5 variant expression through ATF4, thereby enhancing glutamine utilization and reinforcing metabolic plasticity [159]. These findings indicate that ISR activation does not merely suppress global translation but actively reshapes metabolic substrate flow to sustain redox equilibrium. This adaptive coupling creates a metabolic–ISR resilience axis. NADPH flux preserves lipid homeostasis and prevents ferroptotic collapse, while eIF2α–ATF4 signaling increases the transcriptional capacity to regenerate antioxidant systems. When one arm is partially impaired, the other compensates. Consequently, therapeutic strategies targeting only metabolic pathways or only ISR signaling frequently encounter resistance due to circuit redundancy. Dual interference of this axis provides a mechanistically rational approach. Disruption of NADPH production weakens glutathione recycling and sensitizes cells to lipid peroxidation. Concurrent inhibition of eIF2α phosphorylation or ATF4 translation blocks compensatory upregulation of antioxidant genes and amino acid transporters. The resulting collapse of reductive buffering capacity promotes ferroptotic vulnerability and limits adaptive plasticity. Importantly, recent comprehensive analyses of ISR signaling in cancer emphasize that ISR activity functions as a context-dependent switch between survival and death, depending on stress magnitude and duration [160,161,162]. Therefore, therapeutic modulation should aim not merely to inhibit ISR globally, but to shift its output from adaptive redox maintenance toward pro-death signaling under metabolically compromised conditions. In breast cancer, particularly metabolically flexible TNBC, coordinated targeting of NADPH flux and ISR signaling may represent a strategy to dismantle redox buffering while preventing translational adaptation. Such an approach aligns with the broader concept of circuit-guided therapy, in which metabolic and stress signaling networks are co-disrupted to prevent compensatory escape.

### 5.3. Immune–Metabolic ISR Coupling

The adaptive and innate immune landscapes within the tumor microenvironment are profoundly shaped by metabolic reprogramming and stress signaling. In breast cancer, particularly in metabolically constrained niches characterized by hypoxia, nutrient limitation, and oxidative pressure, immune cell function becomes tightly linked to intracellular redox state and translational stress control. The integrated stress response therefore emerges not merely as a tumor-intrinsic survival pathway but as a regulatory interface that coordinates immune metabolism, inflammatory tone, and antitumor immunity. Metabolic reprogramming is a fundamental determinant of immune cell fate and function. Activated effector T cells and proinflammatory macrophages preferentially shift toward glycolysis, while regulatory and memory subsets rely more heavily on oxidative phosphorylation and mitochondrial integrity. This metabolic plasticity governs cytokine production, antigen presentation capacity, and cytotoxic effector functions. Comprehensive analyses of immune–metabolic regulation demonstrate that glycolytic flux, mitochondrial reactive oxygen species production, and tricarboxylic acid cycle remodeling collectively determine inflammatory versus tolerogenic immune states [163,164,165]. Within the tumor microenvironment, competition for glucose and amino acids disrupts this balance, frequently driving T-cell exhaustion and suppressive myeloid phenotypes. The integrated stress response intersects with these immune–metabolic circuits at multiple levels. Engagement of pattern recognition receptors, recognition of damage-associated molecular patterns, and accumulation of misfolded proteins converge on eIF2α phosphorylation and ATF4 activation. This signaling axis not only reprograms tumor cell metabolism but also modulates cytokine production and antigen processing in immune cells. Conceptual frameworks integrating innate immune recognition and stress signaling emphasize that PRR-driven stress responses activate autophagy, mitochondrial reactive oxygen species generation, and inflammasome signaling in parallel [166,167]. Consequently, ISR activation can amplify inflammatory signaling under certain contexts while promoting adaptive tolerance in others, depending on metabolic substrate availability and stress intensity. In breast cancer, estrogen receptor signaling further modulates immune exclusion and inflammatory tone. ERα-driven transcriptional programs suppress interferon signaling pathways, dampen antigen presentation, and influence myeloid cell recruitment [168]. These effects are metabolically conditioned, as ERα signaling intersects with nuclear factor κB and mitochondrial pathways that regulate redox balance. In nutrient-deprived tumors, ISR activation may reinforce this immune suppression by limiting global protein synthesis while selectively enhancing stress-adaptive transcripts that favor immune tolerance. Neutrophil and myeloid cell crosstalk adds an additional metabolic dimension to this axis. Sterile inflammation driven by extracellular DNA, mitochondrial damage, and purinergic signaling alters T-cell differentiation and regulatory T-cell stability [169]. ATP release within the tumor microenvironment and its enzymatic conversion to adenosine by CD39 and CD73 critically modulate T-cell receptor signaling thresholds and cytokine production, thereby linking metabolic byproducts to adaptive immune polarization [169,170]. Adenosine-rich niches suppress effector T-cell activation while favoring regulatory T-cell stability, establishing a metabolically enforced immune tolerance state. Under persistent oxidative and nutrient stress, activation of the integrated stress response further constrains translational output and reshapes amino acid metabolism, which may reinforce regulatory phenotypes and attenuate effector cytokine programs [166]. Collectively, these findings support a model in which immune metabolism and ISR signaling operate as coordinated regulatory modules rather than independent processes. Metabolic stress within tumor cells induces ISR activation that alters cytokine expression patterns and nutrient consumption profiles, thereby indirectly influencing immune cell functionality [163]. Concurrently, immune cells subjected to metabolic competition activate cell-intrinsic stress pathways that shape effector differentiation, exhaustion trajectories, and inflammatory amplitude. The resulting bidirectional feedback loop stabilizes an immune–metabolic equilibrium that frequently favors tumor persistence. From a therapeutic perspective, coordinated modulation of this axis offers advantages over isolated pathway inhibition. Disruption of tumor NADPH buffering capacity sensitizes cells to lipid peroxidation and ferroptotic vulnerability, whereas attenuation of maladaptive ISR signaling may prevent compensatory antioxidant reinforcement. In parallel, restoration of metabolic competence in effector T cells and limitation of adenosine-mediated suppression could reinvigorate antitumor immunity [163,164]. Thus, intervention at the level of immune–metabolic ISR coupling may recalibrate the tumor microenvironment toward sustained cytotoxic inflammation rather than checkpoint blockade alone. In this framework, immune–metabolic ISR coupling represents a systems-level regulatory node integrating redox stress, nutrient sensing, translational control, and inflammatory signaling. Targeting this coordinated network may provide a mechanistically grounded strategy to overcome immune resistance in metabolically adaptive breast cancer.

## 6. Translational Roadmap

### 6.1. Chronic Stress-Induced Adaptive Circuits and Metastatic Memory

**Figure 2:**
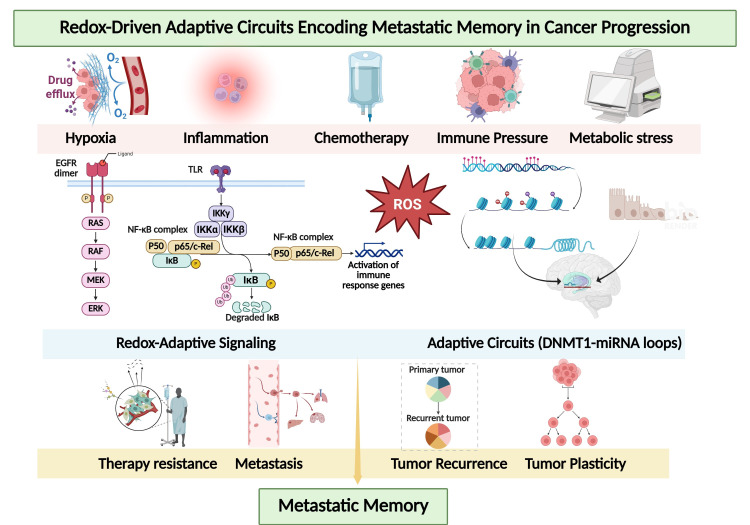
Redox-driven adaptive circuits encoding metastatic memory in cancer progression. Chronic tumor-associated stresses, including hypoxia, inflammation, chemotherapy-induced stress, immune pressure, and metabolic perturbations, elevate intracellular ROS levels, representing upstream stress inputs that initiate the adaptive circuit. ROS activate multiple redox-responsive signaling pathways, including NF-κB, NRF2, MAPK, PI3K/AKT, and HIF signaling networks, constituting the intracellular redox-adaptive signaling layer that integrates stress responses. Sustained activation of these pathways promotes epigenetic remodeling through DNMTs, TETs, and microRNA-mediated regulatory circuits, forming a regulatory layer that stabilizes transcriptional states via feedback and feedforward loops, thereby stabilizing transcriptional programs associated with EMT, cancer stemness, and cellular plasticity. These interconnected signaling and epigenetic feedback loops establish a persistent adaptive state referred to as metastatic memory, representing a circuit-encoded, stress-adapted tumor state, which contributes to therapy resistance, metastatic dissemination, tumor recurrence, and lineage plasticity during cancer progression—the phenotypic output layer of the adaptive circuit framework. Abbreviations: DNA methyltransferase (DNMT); epithelial–mesenchymal transition (EMT); Hypoxia-inducible factor (HIF); Mitogen-activated protein kinase (MAPK) MicroRNA (miRNA); Nuclear factor erythroid 2–related factor 2 (NRF2); Nuclear factor kappa B (NF-κB); Phosphoinositide 3-kinase/protein kinase B (PI3K/AKT); reactive oxygen species (ROS); Ten-eleven translocation enzyme (TET). Created in BioRender. Park, M.N. (2026) https://BioRender.com/rds57iy, accessed on 24 March 2026.

Cancer progression is increasingly recognized not merely as a consequence of genetic mutations but as the outcome of adaptive cellular circuits formed under persistent environmental stress. Chronic exposure to oxidative, inflammatory, and metabolic stress reshapes intracellular signaling networks, enabling tumor cells to transition from transient stress responses to stabilized adaptive states. In this context, ROS function not only as damaging agents but also as regulatory signals that progressively rewire transcriptional and epigenetic programs governing tumor plasticity and survival [171]. Accumulating evidence indicates that prolonged redox imbalance promotes the formation of self-reinforcing regulatory circuits involving redox-sensitive transcription factors, epigenetic regulators, and non-coding RNAs. These circuits frequently include reciprocal feedback loops between microRNAs (miRNAs) and DNA methyltransferases (DNMTs), which function as bistable regulatory modules capable of maintaining stable gene expression states even after removal of the initiating stimulus. Such DNMT–miRNA feedback architectures operate as epigenetic memory units, enabling cancer cells to preserve phenotypic traits associated with EMT, stemness, immune evasion, and therapeutic resistance over extended periods [172]. Within the tumor microenvironment, persistent oxidative signaling further integrates with adhesion dynamics and extracellular communication systems. Recent conceptual frameworks propose that redox signaling intersects with adhesion molecules and exosome-mediated communication to form closed-loop regulatory systems that stabilize metastatic phenotypes. For example, ROS-driven EMT activation can be reinforced by adhesion signaling pathways such as AXL–FAK/Src, while selective exosomal cargo—including regulatory miRNAs and immune-modulatory molecules—propagates these adaptive signals across tumor and stromal cell populations. Through such multilayered interactions, transient oxidative pulses can be converted into durable phenotypic programs that sustain metastatic competence [52]. These observations suggest that cancer cells can develop what may be described as “metastatic memory,” a systems-level property whereby chronic stress conditions encode persistent adaptive states within regulatory networks. Unlike conventional genetic mutations, metastatic memory is maintained through dynamic yet stable circuit architectures involving redox signaling, epigenetic regulation, metabolic adaptation, and intercellular communication. Consequently, the persistence of these adaptive circuits may explain why tumors frequently regain aggressive phenotypes after initial therapeutic responses [172]. Recognizing metastatic memory as a circuit-level phenomenon has important translational implications. Conventional anticancer strategies typically aim to suppress individual oncogenic pathways; however, such approaches may fail to disrupt the broader adaptive networks that stabilize malignant phenotypes. Effective therapeutic strategies may therefore require network-level interventions capable of destabilizing these stress-adaptive circuits, restoring redox balance, and reprogramming the regulatory architecture that sustains tumor plasticity and metastasis. From this perspective, the development of physiologically relevant experimental models capable of recapitulating chronic stress-driven adaptive states becomes a critical priority for translational oncology. Persistent redox stress drives the formation of interconnected signaling and epigenetic regulatory circuits that stabilize malignant cellular states. Through the coordinated activation of redox-responsive pathways and epigenetic memory modules, cancer cells acquire long-lasting adaptive traits that promote metastatic competence and therapy resistance (Figure 2). Importantly, redox-adaptive circuits are closely integrated with oncogenic receptor signaling pathways that regulate tumor cell survival and plasticity. The NRF2 pathway acts as a master regulator of antioxidant defense and promotes the expression of detoxification enzymes and ferroptosis-protective factors such as GPX4 and SOD2. Sustained activation of NRF2 signaling has been associated with increased invasive capacity, epithelial–mesenchymal transition, and therapeutic resistance in multiple cancer models [153]. In parallel, receptor tyrosine kinase signaling mediated by members of the epidermal growth factor receptor family, including EGFR and HER2, activates downstream PI3K–AKT and MAPK pathways that support tumor cell proliferation and survival. Increasing evidence suggests that NRF2-dependent antioxidant programs cooperate with EGFR/HER2 signaling networks to maintain cancer stem cell populations and reinforce redox buffering capacity under therapeutic stress [154]. Cancer stem-like cells characterized by elevated aldehyde dehydrogenase activity and enhanced NRF2 signaling exhibit reduced intracellular reactive oxygen species levels and increased tolerance to chemotherapy-induced oxidative damage. This redox-protected stem-like state facilitates tumor recurrence and metastatic dissemination by allowing therapy-resistant subpopulations to persist within the tumor ecosystem [154,155].

### 6.2. Preclinical Modeling

Preclinical models that credibly anticipate clinical resistance in breast cancer need to reproduce two properties that conventional monolayer culture routinely erases. First, they must preserve spatially structured microenvironmental stress, including diffusion-limited hypoxia and nutrient gradients, because these constraints are the physiological context in which redox buffering, integrated stress response (ISR) signaling, and drug-tolerant persistence are selected. Second, they must preserve state diversity and state-transition capacity, because the resistant phenotype is frequently a dynamic redistribution across accessible cell states rather than a fixed clonal trait.

#### 6.2.1. 3D Spheroid Models

Three-dimensional (3D) spheroid systems provide a pragmatic first layer of this translational stack because they recapitulate core features that directly map onto the adaptive circuits emphasized in Section 3 and Section 4. In triple-negative breast cancer (TNBC), spheroid culture increases epithelial–mesenchymal transition (EMT)-associated protein expression and produces higher drug resistance than two-dimensional (2D) culture, with systematically higher IC_50_ values observed for cytotoxic agents in 3D compared with 2D conditions [173,174]. Large-panel comparisons across TNBC cell lines further indicate that 3D culture generally increases resistance to anthracyclines, platinum, and taxanes relative to 2D assays, supporting the premise that 3D sensitivity profiles can better approximate in vivo drug tolerance phenotypes [175,176]. Importantly, these studies also show that drug class matters, because correlations between 2D and 3D sensitivity can be stronger for DNA-damaging agents than for mitotic poisons, implying that replication stress and DDR engagement may be more faithfully captured by certain model contexts and endpoints than others [175].

#### 6.2.2. Patent-Derived Models (PDOs/PDXOs)

Patient-derived organoids (PDOs) and patient-derived xenograft organoids (PDXOs) represent the next translational layer because they retain patient-specific architecture and, in many implementations, preserve clinically relevant response heterogeneity. Reviews of TNBC 3D modeling emphasize that PDO and PDXO platforms can reproduce tumor features and drug response patterns more faithfully than 2D culture while providing a tractable system for drug testing and mechanistic dissection [177].

#### 6.2.3. Functional Readouts for Circuit Validation

Model construction should therefore be explicitly aligned to the readouts that report on your conserved modules. In spheroids and organoids, redox buffering can be quantified by ROS dynamics and antioxidant capacity proxies, while NADPH homeostasis can be assessed through NADPH/NADP^+^ measurements or flux-informed surrogate markers, and ferroptosis liability can be indexed by lipid peroxidation and GPX4 dependence. Plasticity can be monitored through EMT-state markers and functional invasion readouts that are enhanced after spheroid formation and re-plating, consistent with EMT-linked state plasticity that emerges under 3D constraints [173,174,178]. DDR adaptation can be captured by replication stress signaling outputs and functional HR measures such as RAD51 foci, which are increasingly positioned as functional biomarkers for stratification and therapy response prediction in breast cancer [150].

#### 6.2.4. Tumor Microenvironment Integration

Because resistance is frequently co-produced by tumor–stroma interactions, incorporation of stromal components should be treated as a design requirement when the goal is to test immune–metabolic ISR coupling or microenvironment-reinforced plasticity. Evidence from the 3D model literature indicates that adding cancer-associated fibroblasts (CAFs) can reproduce stromal contributions to EMT, anti-apoptotic signaling, and therapy resistance that are not observed in 2D culture, and that CAFs can recruit macrophages and enhance resistance in breast cancer spheroids [179]. Co-culture systems incorporating CAFs and immune cells provide a practical platform to evaluate how tumor microenvironmental components function as reinforcing modulators of adaptive stress-response circuits, enabling experimental dissection of stromal and immune contributions to therapy resistance.

#### 6.2.5. Translational Application to Phytotherapeutics

Within this framework, phytotherapeutic testing becomes more credible when performed in model tiers that preserve the adaptive circuit being targeted. A phytotherapeutic candidate proposed to destabilize redox buffering and plasticity simultaneously should demonstrate activity in 3D contexts that display increased EMT signatures and drug resistance relative to 2D culture, because that context better operationalizes the clinically relevant resistant state-space [173]. Similarly, a candidate proposed to disrupt metabolic resilience should be evaluated in 3D settings, where metabolic and transporter heterogeneity, including upregulation of efflux machinery and altered metabolic priorities, are more representative of in vivo-like resistance biology [179].

### 6.3. Organoid Systems

Patient-derived organoid (PDO) systems have emerged as translationally robust platforms that complement early-phase clinical trial design by preserving tumor architecture, clonal heterogeneity, and molecular-subtype-specific signaling networks. Unlike conventional two-dimensional cultures, breast cancer organoids maintain luminal–basal hierarchy, receptor expression patterns, and genomic alterations reflective of the original tumor, thereby providing a functional ex vivo surrogate of patient-specific therapeutic response.

#### 6.3.1. Role in Early-Phase Clinical Translation

In early-phase and window-of-opportunity trials, organoid systems serve as biologically informative preclinical filters analogous to phase 0 pharmacodynamic validation strategies [180,181]. Similarly, PDO-based drug sensitivity testing enables rapid functional assessment of candidate therapeutics within clinically relevant timeframes, particularly between diagnosis and initiation of standard-of-care therapy. This strategy enhances cost-efficiency and reduces late-phase attrition—an issue well-recognized in oncology drug development [180].

#### 6.3.2. Integration with Molecular Profiling

Contemporary translational oncology increasingly advocates integration of biospecimen-based mechanistic validation into early clinical development pipelines [182]. Organoid systems fulfill this need by allowing coupling of drug response profiling with transcriptomic and epigenomic interrogation, thereby facilitating mechanistic biomarker discovery.

#### 6.3.3. Subtype-Specific Functional Stratification

In breast cancer, subtype-specific vulnerabilities (e.g., HR^+^, HER2^+^, triple-negative) demand functional stratification beyond static genomic classification. POD platforms allow assessment of drug sensitivity across these subtypes, providing a functional layer that complements genomic profiling.

#### 6.3.4. Microenvironment-Integrated Organoid Systems

Incorporation of stromal and immune components into organoid co-culture systems further refines their translational relevance. Such systems are particularly important for evaluating immunotherapy combinations and microenvironment-modulating strategies in early-phase trials [183].

#### 6.3.5. Translational Significance

Breast cancer-derived organoid systems provide a mechanistically aligned bridge between laboratory discovery and window-of-opportunity clinical designs. By integrating functional drug testing, molecular profiling, and early PD readouts, organoids enhance the biological precision of therapeutic prioritization and support more rational early-phase clinical development.

### 6.4. Biomarker Validation

A clinically actionable biomarker framework for chemoresistance in breast cancer should prioritize readouts that report circuit activity rather than single driver mutations, because the phenotypes discussed in Section 3 and Section 4 arise from state transitions that can be sustained without additional genomic events. In practice, this means that candidate biomarkers should capture the degree of redox buffering, replication stress tolerance, ISR engagement, and EMT–stemness plasticity within treatment-exposed tumors, and should remain interpretable across ER-positive disease, HER2^+^ disease, and TNBC.

#### 6.4.1. Redox–Ferroptosis Biomarkers

For the redox–ferroptosis axis, the most mature evidence supports a predictive role for the ACSL4–GPX4 balance in patients treated with neoadjuvant chemotherapy. In a clinical cohort study, higher ACSL4 expression was associated with better pathological response, whereas higher GPX4 expression was linked to poorer response and inferior outcomes, supporting the interpretation that ferroptosis-permissive tumors are more likely to achieve deep chemotherapy responses [80,184]. This clinical relationship is mechanistically coherent with tissue and cell line observations showing that GPX4 expression in breast cancer correlates with system xCT components and defines dependency on the cystine import program for ferroptosis protection, which provides a mechanistic rationale for stratifying tumors that may respond to ferroptosis-inducing combinations [185,186]. Accordingly, the ACSL4–GPX4 axis should be interpreted as a circuit-level indicator of the ferroptosis threshold rather than as isolated antioxidant markers.

#### 6.4.2. SLC7A11 as a Redox–Immune Biomarker

SLC7A11 represents an additional biomarker linking redox buffering to the immune context. Clinical analyses indicate that elevated SLC7A11 expression in non-metastatic breast cancer is associated with poorer survival outcomes and reduced cytotoxic immune infiltration, alongside increased expression of immune checkpoint-related markers [187]. An independent study also linked SLC7A11 mRNA and protein expression with higher grade and subtype-associated copy number changes, which supports its utility as a clinically measurable marker that reflects aggressive biology and potentially higher tolerance capacity under stress [188]. SLC7A11 is most informative when interpreted in combination with GPX4, NADPH-associated markers, and plasticity-associated signatures, as isolated elevation may reflect multiple upstream stress conditions, including oxidative stress and metabolic cysteine demand.

##### ISR-Related Biomarkers

ISR activity can be operationalized using phospho-eIF2α and ATF4-associated transcriptional programs. In HER2^+^ breast cancer, activation of the PKR–eIF2α axis has been associated with ATF4-dependent tumor-suppressive signaling and improved trastuzumab response, with supporting clinical correlations indicating that eIF2α phosphorylation may predict therapeutic sensitivity in this context [189,190]. In contrast, in TNBC, ATF4 expression has been associated with aggressive phenotypes and poorer survival outcomes, particularly when coupled with TGFβ signaling and plasticity-associated programs [191]. These findings indicate that ISR biomarkers exhibit context-dependent behavior and should be interpreted alongside tumor subtype, EMT state, and metabolic signatures.

##### Multivariable Biomarker Panel Strategy

A practical validation strategy is to construct multivariable biomarker panels integrating ferroptosis threshold, cystine import, ISR activity, and plasticity status. A minimal panel may include GPX4 and ACSL4 protein expression, SLC7A11 levels, and phospho-eIF2α- or ATF4-associated signatures, with EMT–stemness markers serving as modifiers of state-transition capacity. Such integrated panels can be initially evaluated in retrospective cohorts and subsequently translated into prospective validation settings, including window-of-opportunity trials. These biomarkers can be operationalized using clinically applicable platforms, including immunohistochemistry (IHC) for protein expression, transcriptomic profiling for pathway signatures, and, where feasible, proteomic or phospho-protein analyses for dynamic signaling states. These biomarker-driven strategies are consistent with recent clinical syntheses emphasizing dynamic biomarker-guided treatment adaptation and personalized therapeutic sequencing in breast cancer management [192].

### 6.5. Clinical Trial Design

The successful clinical translation of redox–ISR–plasticity circuitry requires trial designs that are aligned with biological state rather than histologic subtype alone. Conventional phase II–III paradigms, which evaluate treatment effects across broadly defined ER-positive, HER2^+^, or triple-negative cohorts, are insufficient to interrogate adaptive stress programs that are dynamically engaged during therapy exposure. Because the resistance phenotypes described in earlier sections arise from circuit-level buffering of oxidative stress, translational arrest, and phenotypic plasticity rather than from single genomic alterations, clinical trial designs must be structured to detect modulation of these stress-adaptive states. In this context, integrating metabolic and redox-associated biomarkers into clinical trial design is further supported by emerging evidence highlighting mitochondrial and metabolic reprogramming as central drivers of therapeutic resistance and immune modulation in cancer [193].

#### 6.5.1. Biomarker-Based Stratification

A rational approach is to incorporate prospective biomarker stratification based on ferroptosis buffering capacity and ISR activation state at baseline. Tumors characterized by high GPX4 expression, elevated SLC7A11, and transcriptional evidence of NADPH-dependent redox maintenance represent a ferroptosis-buffered phenotype that may be intrinsically less responsive to cytotoxic chemotherapy but potentially more vulnerable to strategies that disrupt cystine import or lipid peroxide detoxification. Conversely, tumors exhibiting high ACSL4 and low GPX4 may be closer to a ferroptotic threshold and more likely to achieve deep responses with oxidative or DNA-damaging therapies. Embedding these biomarkers into eligibility or stratification criteria enables transition from histology-driven enrollment to circuit-informed enrichment strategies.

#### 6.5.2. Enrichment and Validation Strategies

Prospective biomarker-driven enrichment and integrated validation strategies are well established within precision oncology trial methodology and provide a statistically rigorous framework for evaluating targeted therapeutic strategies [194,195,196].

#### 6.5.3. Adaptive Signature-Based Trial Design

Given the dynamic nature of stress-response circuitry, adaptive signature-based trial designs are particularly relevant. These designs enable development and validation of predictive classifiers within a single randomized study while maintaining statistical control [197,198]. A multivariable classifier integrating GPX4-, SLC7A11-, phospho-eIF2α-, and ATF4-associated transcripts and EMT-related markers can be developed in a training subset and prospectively validated in an independent cohort to assess differential treatment response across circuit-defined subgroups [197,198]. Such approaches allow integration of multiple stress-adaptive signals that may not be sufficiently predictive when considered individually.

##### Window-of-Opportunity Design

Short pre-operative window-of-opportunity studies provide a translational bridge between mechanistic hypotheses and clinical validation. Administration of redox- or metabolic-targeting agents prior to surgery enables paired tumor sampling to assess pharmacodynamic engagement in treatment-naïve tumors. Relevant readouts include lipid peroxidation markers, GPX4 expression, ATF4 target genes, and plasticity-associated transcripts. These studies function as pharmacodynamic validation platforms that support subsequent phase II–III development [199].

##### Multivariable Modeling and Statistical Integration

Prospective integration of multivariable modeling is required for robust biomarker validation. Cox proportional hazards analyses incorporating ferroptosis indices, ISR activation scores, and established clinicopathologic variables can determine whether circuit-level biomarkers independently predict response, recurrence, or survival. Methodologic guidance emphasizes pre-specification of classifier algorithms, control of multiplicity, and separation of prognostic versus predictive endpoints to ensure interpretability and regulatory validity [200,201].

##### Translational Implication

These approaches support a shift from mutation-centric trial design toward state-based adaptive frameworks that reflect redox regulation, translational control, metabolic adaptation, and phenotypic plasticity. Clinical evaluation of therapies targeting metabolic adaptation should therefore integrate biomarker enrichment, adaptive classifier development, and pharmacodynamic validation to assess both clinical efficacy and modulation of stress-adaptive tumor states. An illustrative trial workflow may include baseline tumor biopsy with comprehensive biomarker profiling of GPX4, ACSL4, SLC7A11, and phospho-eIF2α/ATF4 signatures and EMT markers, followed by patient stratification into circuit-defined subgroups, such as ferroptosis-buffered versus ferroptosis-permissive or ISR-high versus ISR-low states. Patients can then be assigned to mechanism-aligned treatment arms, including redox-modulating combinations or standard chemotherapy, with on-treatment pharmacodynamic validation performed using paired tumor samples or liquid biopsy approaches to confirm circuit disruption. Clinical endpoints should include pCR, progression-free survival (PFS), and OS.

## 7. Discussion

Breast cancer chemotherapy resistance emerges as a systems-level property of a dynamically reconfiguring tumor ecosystem rather than as a fixed consequence of clonal genetic evolution. Single-cell and longitudinal analyses across breast cancer subtypes demonstrate that therapeutic stress reshapes transcriptional state distributions, enabling redistribution within a constrained yet plastic phenotypic landscape [106,110,129,130]. These findings support the concept that resistance frequently arises through state transition rather than exclusive expansion of pre-existing resistant clones. Mechanistically, multiple adaptive axes converge to stabilize stress-tolerant phenotypes. Activation of the integrated stress response under hypoxia, mTOR inhibition, or cytotoxic therapy selectively enhances translation of stemness- and EMT-associated transcripts, reinforcing plastic identity programs [131,132]. Inflammatory reinforcement through JAK–STAT signaling further stabilizes mesenchymal and chemoresistant states in breast cancer models [133,134]. In parallel, metabolic rewiring increases NADPH production and enhances reactive oxygen species buffering capacity, thereby maintaining redox permissiveness under therapeutic pressure [135,136]. Stromal remodeling and immune-context alterations additionally support persistence of residual populations [137]. Taken together, these converging mechanisms indicate that therapeutic intervention functions not only as a selective filter but as a driver of regulatory reconfiguration within the tumor ecosystem [76,96,99,100,101]. Suppression of a dominant oncogenic pathway can eliminate proliferative compartments while leaving intact the epigenetically permissive transitions that regenerate them [104,105]. Without simultaneous disruption of stress-response signaling, metabolic buffering, and inflammatory stabilization circuits, state redistribution remains feasible, and recurrence becomes probable [133,138]. This systems-level view helps reconcile clinical observations in ER^+^, HER2^+^, and triple-negative breast cancers, where initial response is frequently followed by adaptive relapse despite effective target inhibition. Resistance therefore reflects constrained yet reversible navigation across a bounded phenotypic manifold rather than a unidirectional genetic trajectory. Importantly, emerging evidence suggests that chronic therapeutic stress can establish self-reinforcing regulatory circuits that function as a form of cellular stress memory. Once activated, these circuits integrate redox regulation, inflammatory signaling, metabolic buffering, and epithelial–mesenchymal plasticity into a stabilized regulatory state that persists even after removal of the original stress stimulus. Such circuit-level stabilization helps explain why metastatic competence and therapy resistance frequently remain durable despite inhibition of individual signaling pathways. In this framework, metastasis and relapse may reflect the reactivation of previously established adaptive circuits rather than de novo acquisition of resistant mutations. Consequently, effective therapeutic strategies may require disruption of the integrated stress-adaptive circuitry that maintains phenotypic persistence within the tumor ecosystem [52,73,171,172].

### Limitations

Several limitations should be acknowledged. First, much of the mechanistic evidence supporting state transition and therapy-induced reprogramming derives from single-cell transcriptomic analyses and experimental models, which may not fully recapitulate long-term evolutionary dynamics observed in patients. Second, while stress-response and metabolic buffering pathways have been implicated in therapy tolerance, direct clinical validation of circuit-level interventions that constrain phenotypic accessibility remains limited. Third, the state-space framework, although supported by emerging data, represents a conceptual model that requires prospective validation using longitudinal sampling and functional perturbation studies in human tumors. Finally, heterogeneity across breast cancer subtypes may impose subtype-specific constraints on state transitions, and the degree to which plasticity can be therapeutically collapsed may vary depending on lineage architecture and microenvironmental context. Future studies integrating temporal multi-omics profiling with interventional trials will be necessary to test whether restricting state accessibility can meaningfully improve durable response rates. Additional limitations include inter-patient heterogeneity in tumor state composition and the lack of standardized protocols for 3D and organoid-based models, which may affect reproducibility and translational consistency across studies.

## 8. Conclusions

Chemotherapy resistance in breast cancer should not be interpreted solely as the consequence of clonal genetic evolution, but rather as the stabilization of adaptive stress-response circuits that reshape tumor phenotypic landscapes under therapeutic pressure. The SR^3^ network framework proposed in this review integrates steroid receptor signaling, redox buffering, replication stress tolerance, translational plasticity, and metabolic NADPH homeostasis into a unified adaptive architecture that sustains tumor survival during treatment. Importantly, therapeutic interventions themselves function as state-generating perturbations that expand the accessibility of stress-tolerant phenotypes. Consequently, strategies aimed at overcoming resistance must move beyond single-target inhibition toward circuit-guided combination approaches that simultaneously destabilize redox buffering systems, constrain phenotypic plasticity, and disrupt metabolic–ISR resilience axes. Such systems-level therapeutic design may provide a conceptual foundation for preventing adaptive state transitions and achieving more durable control of breast cancer.

## Data Availability

No new data were created or analyzed in this study. Data sharing is not applicable to this article.
